# Long-term renal survival in patients with IgA nephropathy: a systematic review

**DOI:** 10.1080/0886022X.2024.2394636

**Published:** 2024-08-27

**Authors:** Huijian Zhang, Song Ren, Jieqiang Hu, Guisen Li

**Affiliations:** Renal Department, Sichuan Provincial People’s Hospital, University of Electronic Science and Technology of China, Chengdu, China

**Keywords:** IgA nephropathy, renal survival, systematic review, long-term outcomes

## Abstract

The management strategy for IgA nephropathy (IgAN), has undergone constant improvements since the disease entity was first described 50 years ago. However, it is still unknown how these changes affected the long-term renal survival of IgAN patients. We systematically evaluate changes in IgAN renal survival by searching PubMed, Embase, and the Cochrane Library Database of Systematic Reviews from inception to 19 May 2024. We included a large sample of 103076 IgAN cases from 158 studies. Renal survival rates were 94.16% (95% CI: 94.02% to 94.31%), 88.68% (95% CI: 88.48% to 88.87%), and 78.13% (95% CI: 77.82% to 78.43%) at three, five, and ten-year, respectively. Over the past few decades, there haven’t been any sound changes in the 3-year and 5-year renal survival rates. The kidney survival rate in developed countries is higher than in developing countries. Researchers consistently show that while proteinuria < 1.0 g/24 h, renal survival rates increase dramatically. In IgAN, long-term renal survival fluctuated rather than continuously improving over time. Our system review’s findings indicate that supportive care—the most important recommendation for managing IgAN has shown promising results. The long-term outcomes of IgAN could be significantly improved by the latest developed treatment options.

## Introduction

IgA nephropathy (IgAN), a primary glomerular disease defined and reported more than 50 years ago, is one of the leading causes of end-stage kidney disease (ESKD) worldwide [1–5]. A cohort from the International Kidney Biopsy Survey showed that IgAN was the main pathogenic type, particularly in Asia and Europe [6]. IgAN accounted for 36.3% and 52.7%, respectively, of primary glomerular disorders in two large renal biopsy cohort studies in China [3,4].

A nationwide Swedish cohort following 3622 patients(median age, 34.9 years)with IgAN, diagnosed in 1974–2011 for a median follow-up of 13.6 years, reported a 1.53-fold increased risk of death compared with the healthy population, an absolute excess mortality of 3.23 per 1000 person-years and a median reduction in life expectancy of 6 years [7]. A previous study analyzed the cost among 11569 hospitalized adults with IgAN from January 2010 to December 2015 in China, and the median in-hospital cost was about 8000 RMB [8]. In addition, a Japanese study showed the annual costs in 2013 increased from US$1600 for chronic kidney disease (CKD) stage 1 to US$12 700 for CKD stage 5 [9]. Patients with ESKD endured higher annual costs and heavy financial burdens.

Assessment of trends in renal survival could indicate whether changes in treatment strategies have resulted in improved long-term renal outcomes. The therapies most frequently used in patients with IgAN were optimized supportive care, glucocorticoids, and other immunosuppressive, including azathioprine, cyclophosphamide, calcineurin inhibitors (CNIs), and rituximab [10–15]. Recently, the sodium-glucose cotransporter-2 inhibitors (SGLT2i) were added to the first-line regimen for IgAN [16]. Although immunosuppressants are controversial in the treatment of IgAN [10], our previous study [11] and a recent real-world study [17] support the benefits of immunosuppressants in IgAN patients. TESTING found that the frequency of the composite endpoint (40% decrease in eGFR, a need for maintenance dialysis or kidney transplant, or death due to kidney disease) in IgAN was significantly lower in the glucocorticoid group (28.8% in the glucocorticoid group vs 43.1% in the placebo group) [13]. After 36 months, 24h urine protein in the glucocorticoid group did not decrease obviously compared to the control group. And there was no apparent difference in eGFR between the two groups after 96 months. But the reduced dose regimen in TESTING still resulted in significant adverse events [13]. According to the clinical trial, the benefits of glucocorticoids might diminish gradually. Despite the availability of many treatments, poor socioeconomic status may be an important contributor to disease progression to renal failure. Low socioeconomic status could result in delayed diagnosis and suboptimal management [18,19].

What is the impact of these efforts on the clinical outcomes of IgAN? Most previous reviews have focused on epidemiology, clinical manifestation, timing of diagnosis, management and renal prognosis of IgAN [20,21]. But many studies only evaluated the short-term impact of the regimen on the renal outcomes (such as the decline in estimated glomerular filtration rate (eGFR), the change of proteinuria), with less evaluation of long-term renal outcomes. Whether the long-term (5-year, 10-year) outcomes of IgAN also have been changed and improved accompanied by the changes in these treatment regimens? It’s still unclear. We conducted a systematic review to provide estimates of the probability of renal survival in IgAN patients, and to investigate how the trends of clinical outcomes have changed in IgAN over the past several decades. This review attempts to draw attention to the long-term prognosis of IgAN. It was essential to assess the benefit of drugs for long-term renal survival in patients with IgAN. We also explored the impact of socioeconomic status on renal survival by comparing long-term renal survival in IgAN patients from developed and developing countries.

## Materials and methods

### Data sources and search strategy

We performed a systematic review of the published literature on renal survival in patients with IgAN. We followed protocol based on Preferred Reporting Items for Systematic Review and Meta-Analysis guidelines and Meta-analysis of Observational Studies in Epidemiology recommendations [22,23]. This systematic review was registered with PROSPERO (CRD42023414994). We searched the PubMed, Embase and Cochrane Database from inception to 19 May 2024, without language restrictions. The detailed search strategy was showed in Supplementary Appendix A. We also searched the references of these studies and review articles.

### Study selection

Two reviewers (ZHJ and HJQ) independently screened studies based on inclusion and exclusion criteria. The inclusion criteria of our study were: (1) the study design was a randomized controlled trial or a cohort study; (2) all participants were diagnosed as primary IgAN by renal biopsy with estimated glomerular filtration rate (eGFR) ≥15 mL/min/1.73 m^2^; (3) patients did not receive renal replacement therapy; (4) the sample size of IgAN patients ≥ 100, and the median follow-up ≥ 36 months in each study. Exclusion criteria were listed as follows: (1) long-term renal survival was not reported; (2) for the same cohort, except for the report with the longest follow-up, all other duplicate publications were excluded.

### Data extraction and quality assessment

Two reviewers (ZHJ and HJQ) independently completed data extraction forms and applied the Newcastle–Ottawa Quality Assessment Scale to assess the quality of the studies (Supplementary Appendix B). Disagreements were resolved by discussing with an experienced nephrologist (RS). We extracted data including study designs, calendar years of enrollment and length of follow-up, patient characteristics, countries, treatment regimen, and renal survival. Renal survival was defined as the period from diagnosis to composite endpoints. The composite endpoints of renal outcome were the followings: doubling of serum creatinine level, 40% decline in eGFR, or end-stage kidney disease (ESKD). ESKD was defined as eGFR < 15 mL/min/1.73 m^2^ or the initiation of maintenance renal replacement treatment.

### Data synthesis and analysis

In this systematic review, we analyzed renal survival over time and the impact of proteinuria levels on renal survival. We separately analyzed studies from developed countries and developing countries, as classified by the United Nations including 37 developed countries [24]. The raw data of renal survival were reported in the studies in different ways: as Kaplan-Meier curves, as proportions with renal survival at specific follow-up time, and as proportions with renal survival over the period of observation. For the studies in which the renal survival was reported as Kaplan-Meier curves, we used WebPlotDigitizer (version 4.6) developed by Ankit Rohatgi (https://automeris.io/WebPlotDigitizer) to get the data of 3-year, 5-year and 10-year renal survival. Then we calculated average survival rates from different studies at the same midpoint year of patient enrollment, which were weighted by population. The study synthesized 3-year, 5-year and 10-year renal survival with different countries, treatments and eGFR. Analyses were conducted by GraphPad Prism 9 software (San Diego, California, USA), Tableau 2019 (https://www.tableau.com) and IBM SPSS Statistics 26.0. The chi-square test was used to compare renal survival between Asia and Europe and between developed and developing countries. Statistics were considered significant at a two-tailed *P* value (*p* < 0.05).

## Results

### The characteristics of the involved studies

**Figure 1.**
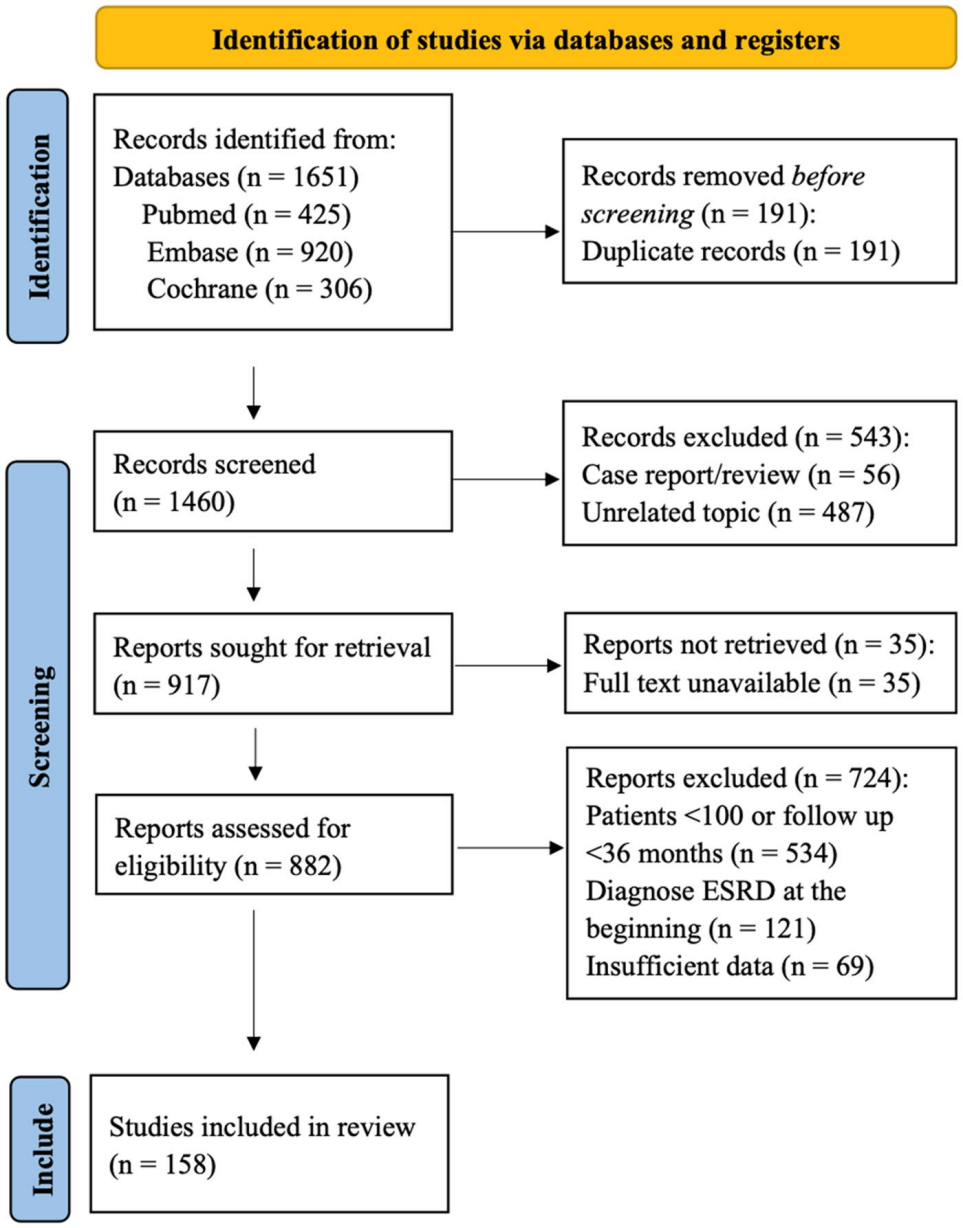
Flow diagram of the literature search and study inclusion.

**Figure 2.**
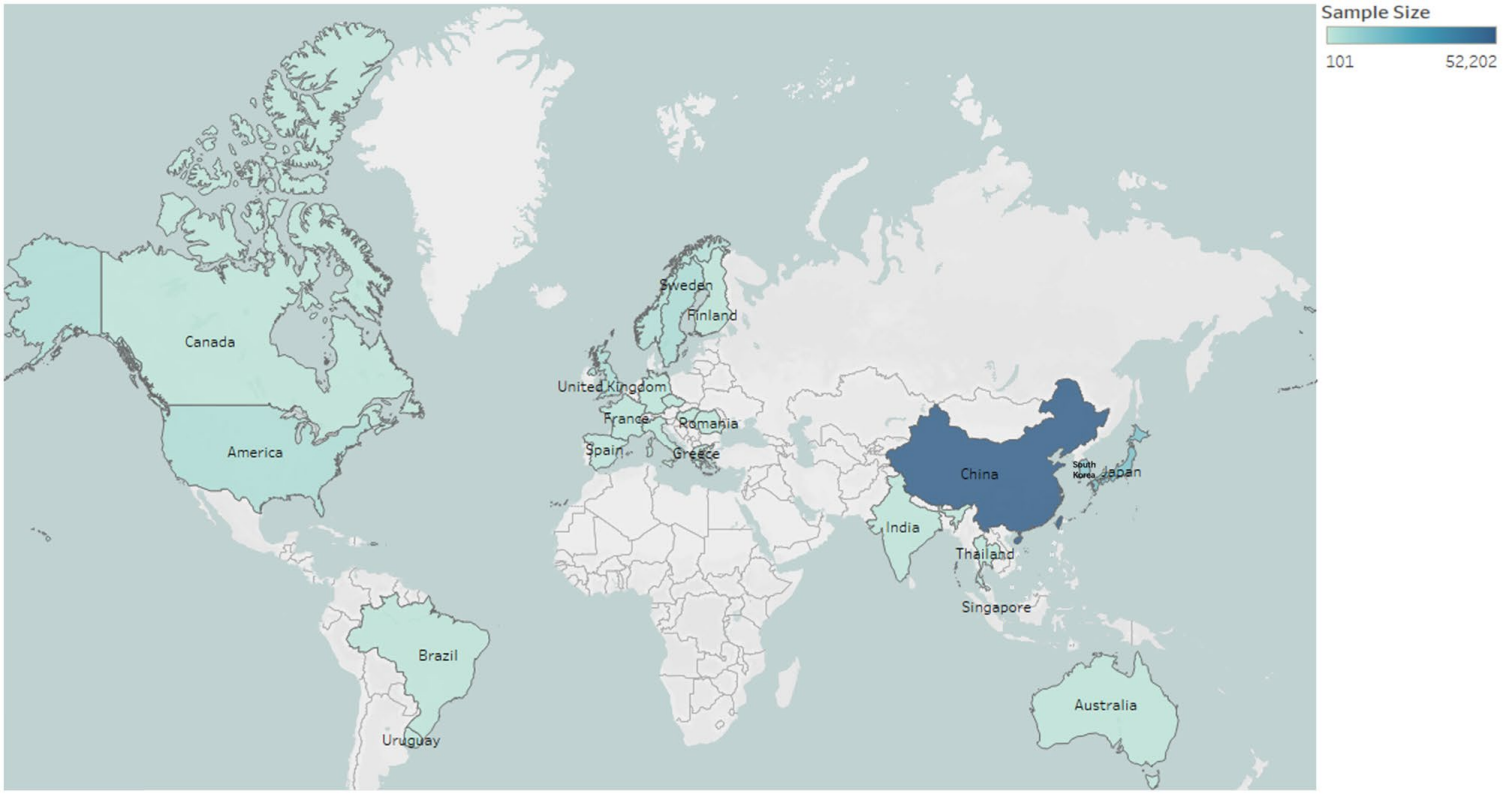
The sample size of IgAN worldwide.

A total of 158 studies, including 103076 IgAN patients, were included in the final analysis. The final dataset included 2 randomized clinical trials and 156 cohort studies published from 1988 to May 2024. 84 studies were selected from developing countries and 74 studies were from developed countries (Figure 1). About 56.8% of patients were male, and the mean age was 35 years. The average eGFR was 77.45 mL/min/1.73 m^2^. The median follow-up was 80 months. Sample sizes in different countries were presented in Figure 2. Detailed information on the included studies was presented in Supplementary Appendix C, Supplementary Appendix D and Supplementary Appendix E.

### Long-term renal survival in all IgAN patients, as well as patients in different countries

**Figure 3. Estimated:**
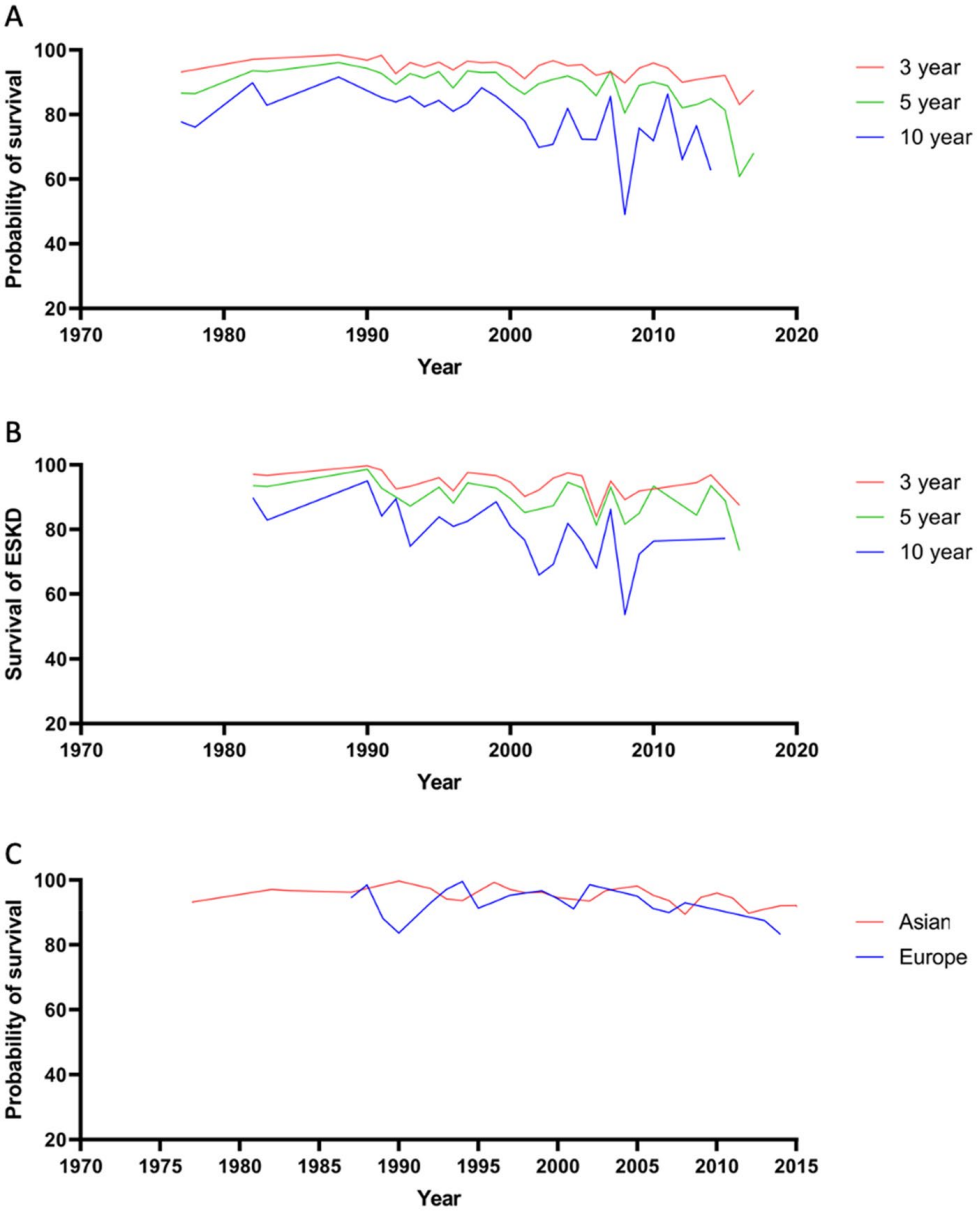
renal survival in IgAN. (A) Estimated renal survival at 3 years, 5 years, and 10 years of IgAN in all countries; (B) Estimated survival rate of ESKD at 3 years, 5 years, and 10 years of IgAN; (C) Estimated renal survival at 3 years of IgAN in Asia and Europe.

In these studies, the 3-year, 5-year and 10-year renal survival were 94.16% [95% confidence interval (CI): 94.02% to 94.31%], 88.68% (95% CI: 88.48% to 88.87%) and 78.13% (95% CI: 77.82% to 78.43%), respectively. In all countries, renal survival for IgAN increased slightly from 1977 to the early 1990s. The 3-year and 5-year renal survival have remained largely unchanged since the beginning of 1990. However, the 10-year renal survival in IgAN has gradually decreased since 1988s (Figure 3A). In addition, the estimated survival rate of ESKD at 3 years, 5 years, and 10 years was shown in Figure 3B. There was no significant change in 3-year renal survival between Asia and Europe in Figure 3C. However, the 3-year median survival in Asia was slightly lower than in Europe (94.60% vs 95.02%, *p* < 0.01). Besides, subgroup analyses were performed after excluding cohorts with enrollment periods longer than 10 years in Supplementary Appendix G.

**Figure 4.**
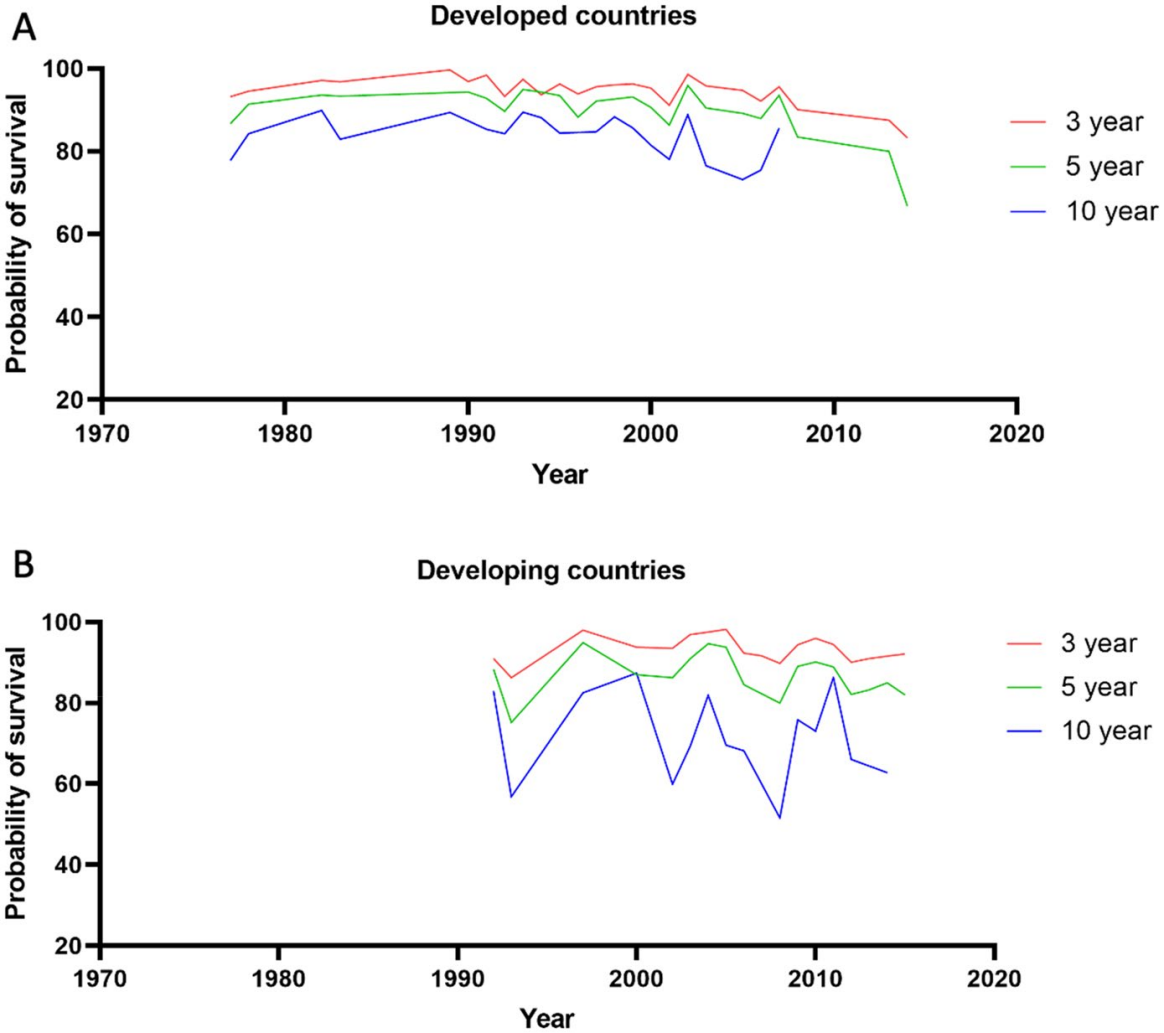
Estimated renal survival at 3 years, 5 years, and 10 years of IgAN in different countries. (A) Estimated renal survival at 3 years, 5 years, and 10 years of IgAN in developed countries. (B) Estimated renal survival at 3 years, 5 years, and 10 years of IgAN in developing countries.

In developed countries, the 3-year, 5-year and 10-year renal survival were 95.31% (95% CI: 95.11% to 95.50%), 91.40% (95% CI: 91.14% to 91.66%) and 82.60% (95% CI: 82.24% to 82.96%), respectively. In the past few years, the renal survival of patients with IgAN was fluctuated, and there was a slight decrease in the early 2010s (Figure 4A).

The data from developing countries in our study started in 1992. In developing countries, the 3-year, 5-year and 10-year survival were 93.06% (95% CI: 92.85% to 93.28%), 86.27% (95% CI: 85.98% to 86.56%) and 71.71% (95% CI: 71.17% to 72.25%), respectively. The 10-year renal survival fluctuated significantly in developing countries (Figure 4B). The 3-year, 5-year and 10-year median survival in developed countries and developing countries showed in Supplementary Appendix H. Renal survival was significantly higher in developed countries than developing countries.

### The impact of proteinuria on long-term renal survival in IgAN

**Figure 5.**
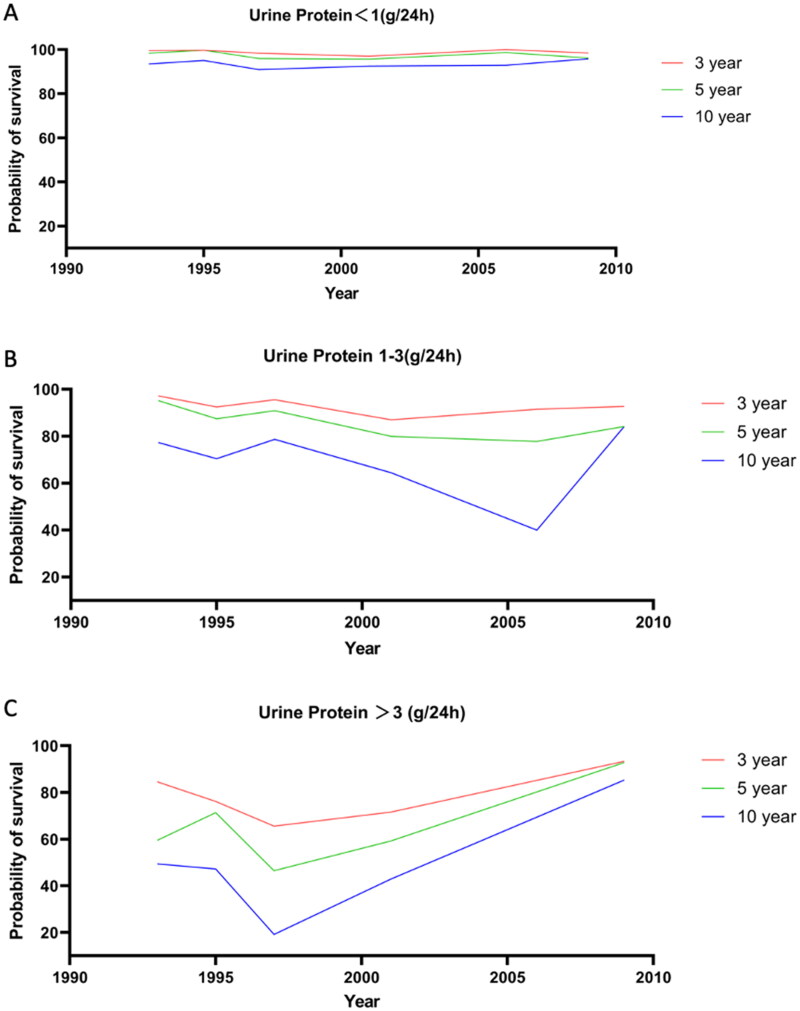
Estimated renal survival at 3 years, 5 years, and 10 years of IgAN with different levels of proteinuria. (A) Estimated renal survival at 3 years, 5 years, and 10 years of IgAN with proteinuria < 1.0 g/24h. (B) Estimated renal survival at 3 years, 5 years, and 10 years of IgAN with proteinuria 1-3 g/24h. (C) Estimated renal survival at 3 years, 5 years, and 10 years of IgAN with proteinuria >3 g/24h.

We also assessed the impact of baseline proteinuria on renal survival in IgAN patients. It was found that when baseline proteinuria was < 1.0 g/24h, the renal survival was high and even the 10-year renal survival was more than 90%. The 3-year, 5-year and 10-year survival were 98.89% (95% CI: 98.55% to 99.23%), 97.62% (95% CI: 97.12% to 98.12%) and 93.85% (95% CI: 92.95% to 94.75%), respectively (Figure 5A).

When the baseline proteinuria was at 1-3 g/24h, the 3-year, 5-year and 10-year renal survival were 89.95% (95% CI 88.83% to 91.09%), 82.80% (95% CI 81.10% to 84.48%) and 72.62% (95% CI 70.48% to 74.80%), respectively. Compared to patients with proteinuria < 1.0 g/24h, renal survival was significantly reduced. Renal survival in patients with IgAN that proteinuria was at 1-3 g/24h was declining until the early 2000s. There has been a gradual increase since 2000. In particular, the 10-year survival increased significantly after 2005 (Figure 5B).

When proteinuria was more than 3 g/24h, the 3-year, 5-year and 10-year renal survival were 87.99% (95% CI: 86.15% to 89.91%), 78.46% (95% CI: 77.12% to 80.87%) and 71.87% (95% CI: 68.31% to 75.49%), respectively. Additionally, we found that the long-term renal survival of patients with IgAN in which proteinuria was >3 g/24h increased gradually after the late 1900s (Figure 5C).

### Long-term renal survival in IgAN patients with different treatment and eGFR

**Figure 6.**
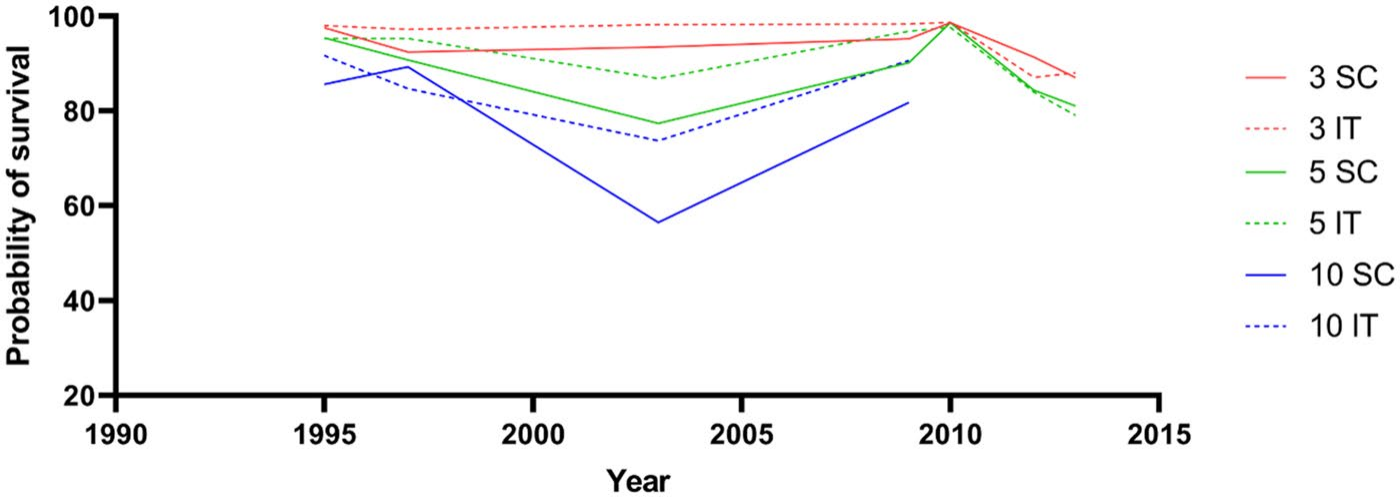
Estimated renal survival at 3 years, 5 years, and 10 years of IgAN with different treatment. SC: supportive care; IT: corticosteroids or immunosuppressive therapy.

When patients were treated with supportive therapy, the 3-year, 5-year and 10-year renal survival were 91.69% (95% CI: 90.57% to 92.79%), 86.97% (95% CI: 85.52% to 88.39%) and 81.05% (95% CI: 79.00% to 83.17%), respectively (Figure 6). When patients were treated with corticosteroids or immunosuppressive therapy, the 3-year, 5-year and 10-year renal survival were 92.83% (95% CI: 91.93% to 93.74%), 89.20% (95% CI: 88.04% to 90.39%) and 86.07% (95% CI: 84.24% to 87.88%), respectively (Figure 6). Immunosuppressive therapy has slightly higher long-term renal survival than supportive care in IgAN.

**Figure 7.**
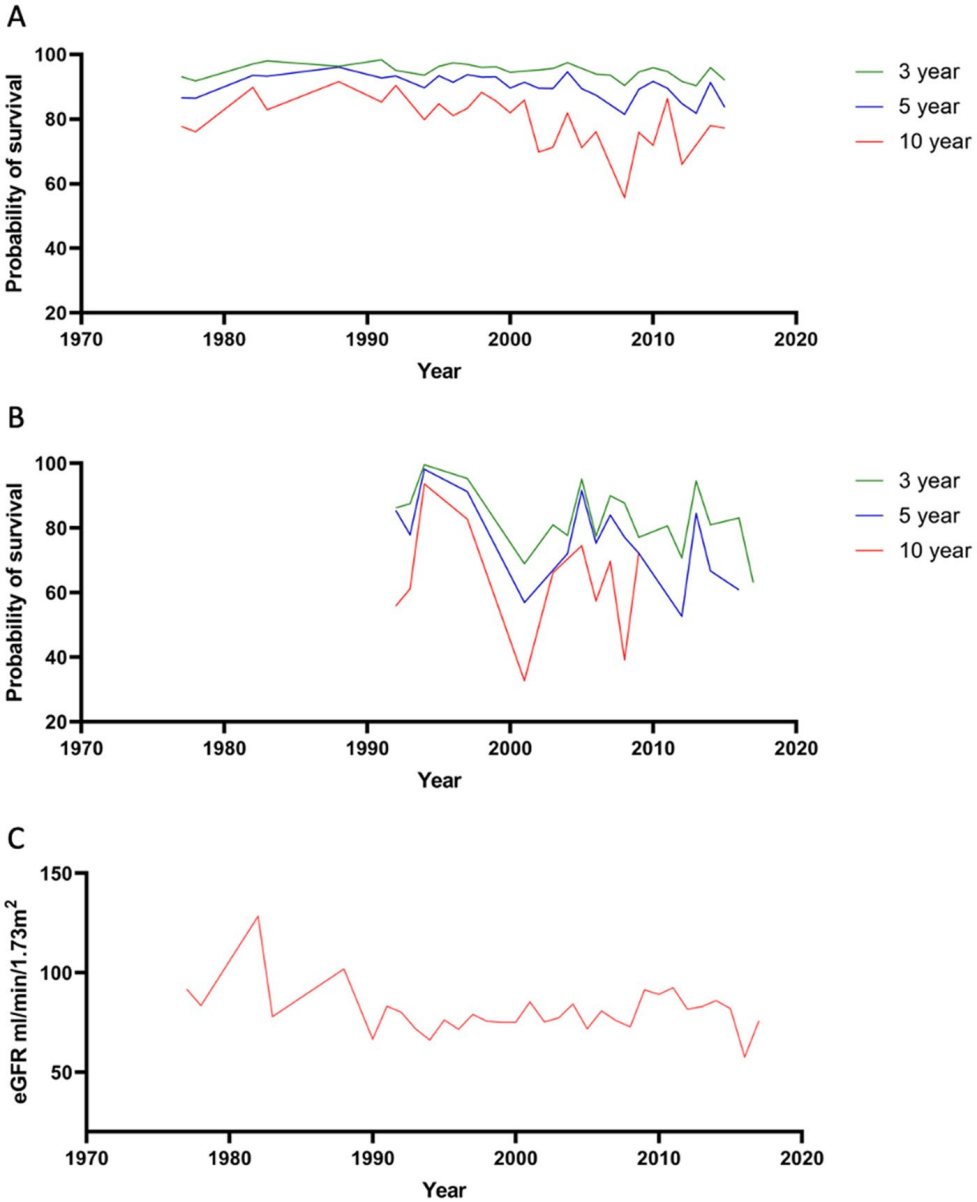
Estimated renal survival at 3 years, 5 years, and 10 years of IgAN with different eGFR. (A) Renal survival IgAN with eGFR ≥60 mL/min/1.73 m^2^; (B) Renal survival IgAN with eGFR < 60 mL/min/1.73 m^2^; (C) Average eGFR in different years.

Renal survival at 3 years, 5 years, and 10 years of IgAN with different eGFR were shown in Figure 7.

## Discussion

To evaluate the trend of long-term survival of IgAN over the past few decades, we included a total of 103076 patients from 158 studies for a systematic review. Although there was an impression that renal survival in IgAN has improved over the past 40 years with the development of medicine, few studies have examined long-term changes in renal survival over different decades. Our key finding of the systematic review was that renal survival in IgAN has remained relatively stable in these years. Long-term outcomes of IgAN were still not significantly improved. In addition, we also investigated the effect of proteinuria on long-term prognosis, we found that while proteinuria was < 1 g/24h, the long-term prognosis of patients with IgAN could be significantly improved, but the long-term renal survival in IgAN patients with high proteinuria (> 3 g/24h) at baseline was still very low.

Only a few studies have compared long-term renal survival in IgAN patients at different stages. A study compared 10-year renal survival in 1981-1995 and 1995-2006. Although the results showed an improvement in renal survival over the past decades, the study was not prospective and there were substantial differences in baseline characteristics between the periods [25]. However, another study reported the probability of ESKD was 12% in the 1st decade from 1976 to 1986 and 19% in the 4th decade from 2008 to 2018, but the patients in the 4th decade were older and had a higher prevalence of hypertension and renal impairment, compared with those in the 1st decade [26]. It is difficult to demonstrate that changes in treatment regimens have a significant impact on the clinical outcomes of IgAN. Therefore, we use the systematic review to demonstrate the changes in IgAN outcomes in recent decades, which can provide a deep insight into the changes in treatment regimens and their impact on clinical outcomes.

Unfortunately, our results revealed that there has been no discernible improvement in the long-term outcome of IgAN, particularly in patients with higher proteinuria. Because of the complexity and heterogeneity of IgAN clinical manifestations, it is challenging to manage IgAN. The cornerstone of IgAN treatment currently remains supportive care [10]. SGLT2 inhibition in DAPA-CKD and EMPA-KIDNEY trials clearly demonstrated renal protection in non-diabetic chronic kidney disease [27,28]. Glucocorticoids have been used in IgAN patients at high risk of progression. Targeted-release formulation of budesonide contributed to a notable decrease in proteinuria and preservation of eGFR compared with placebo for 9 months. However, after 12 months, proteinuria started to rise once more [29]. These studies also suggested that the limited long-term effects of IgAN drugs might be the reason for the failure of IgAN renal survival to improve over time. Sparsentan is a non-immunosuppressive dual antagonist of endothelin and angiotensin receptors. 404 IgAN patients with persistent proteinuria (≥1 g/day) despite full-dose RAASi were randomized to receive sparsentan or irbesartan in the PROTECT trial [30]. Comparing sparsentan and irbesartan at 9 months, the former substantially lowered proteinuria (41% relative reduction between groups) [30]. We desire to see whether sparsentan has the potential to improve long-term renal survival in future studies. Atacicept, which can be combined with BAFF and APRIL, was evaluated in a phase 2 trial of 16 patients with IgAN [31]. Although atacicept improved eGFR, proteinuria did not continue to decline after 24 weeks with atacicept 75 mg [31]. Proteinuria was also effectively reduced by Telitacicept and Sibeprenlimab, but these effects weakened over time [32,33]. Proteinuria < 1.0 g/24h is essential for long-term prognosis, and proteinuria is an excellent indicator for evaluating IgAN treatment which needs to be monitored over a long period. Renal failure, mortality, eGFR, and eGFR slope in IgAN clinical trials are also necessary to evaluate the effectiveness of drugs [34]. In addition, with a number of studies on complement in IgAN, many drugs targeting complement therapy have entered clinical trials, such as Narsoplimab, Iptacopan and so on [35,36]. Although more and more clinical trials have shown that new medications or treatment regimens can improve the clinical outcomes of IgAN patients, the patients in these clinical trials usually were followed up for only a few months or several years (often less than 3 years). It’s difficult to demonstrate that these new medications or treatment regimens can significantly improve the long-term clinical outcomes of IgAN patients. Extending follow-up in clinical trials is imperative to evaluate the potential benefits of these drugs in long-term renal survival. Our results will serve as a point of reference for these clinical trials.

The 3-year, 5-year and 10-year renal survival in developed countries were higher than in developing countries. It could be attributed to many factors, such as the accessibility of drugs, adherence to the regimen, socioeconomic status (SES), as well as the severity of IgAN, etc. A previous study indicated that SES might modify the effect of risk factors on the development of kidney disease [37]. SES in developing countries may delay early diagnosis and timely effective treatment for IgAN patients. Therefore, how to promote the equity in the diagnosis and management of IgAN patients globally still requests the joint efforts of nephrologists.

It is generally recognized that the burden of proteinuria is an important factor affecting the renal survival of IgAN patients [1,2,5,16,38]. Recent evidence supported early changes in proteinuria as a surrogate endpoint for clinical trials in IgAN [34]. Proteinuria was a powerful predictor of IgAN progression [10,38,39]. Our study also demonstrated that patients with higher proteinuria (especially >3 g/24h) had worse renal survival. When proteinuria was >3 g/24h, the long-term survival gradually improved after the late 1990s. It revealed that the active regimens targeting proteinuria would result in the improvement of long-term renal survival for IgAN patients with high proteinuria. A previous study reported that urine protein excretion in IgAN was reduced from 2.49 to 1.12 g/24h between 1994 and 2003 after aggressive therapy and annual ESKD also declined [40]. The improved survival may be related to better control of proteinuria. Another analysis revealed that proteinuria was the most important predictor of the rate of GFR decline. Patients who had proteinuria of more than 3 g/24h and achieved a partial remission (<1 g/24h) had a similar course to patients who had <1 g/24h throughout [39]. These studies have demonstrated the relationship between proteinuria and prognosis in IgAN and the importance of remission.

However, our study had several limitations. First, we did not investigate renal survival beyond 10 years, because few studies have shown that IgAN patients have been followed up for more than 10 years. And the strength of evidence is not very high, because most of the studies are cohort studies. Only two RCT trials fulfilled the criteria for inclusion in this analysis because of their short follow-up period. Second, we did not perform other subgroup analyses that have been associated with renal survival in IgAN, such as age, gender, ethnicity, and hypertension. A majority of studies on long-term survival in IgAN did not explicitly state the race of participants.

## Conclusions

According to our findings, there has not been a rise in long-term renal survival in IgAN over the previous few decades. Our study may serve as a benchmark for evaluating the efficacy of various therapies. Notably, RCT trials are necessary to be followed up to assess the long-term effects of novel drugs on IgAN patients. A significant factor in the long-term prognosis of IgAN was proteinuria. Patients with proteinuria < 1 g/24h had improved long-term renal survival, according to our study. Additionally, developing countries had lower renal survival rates than developed ones. Thus, we should continuously pursue new medications or management strategies that could improve long-term renal survival in IgAN.

## Supplementary Material

Supplementary Materials.doc

## Data Availability

All data relevant to the study are included in the article or uploaded as supplemental information. All data are inside the paper. All data are freely available to any researcher.
