# Supplementary material for: Long-term renal survival in patients with IgA nephropathy: a systematic review
Source: Ren Fail. 2024 Aug 27;46(2):2394636. doi: 10.1080/0886022X.2024.2394636 (PMC11360644; doi:10.1080/0886022X.2024.2394636)
Supplement: Supplementary Materials.doc [file IRNF_A_2394636_SM2218.doc]

Supplementary Materials

Appendix A Database Search Terms

Appendix B Study Quality

Appendix C Characteristics of studies

Appendix D List of Primary Studies Included in the Meta-analysis

Appendix E Sample size of the top20 countries included in the review

Appendix F. PRISMA checklist

Appendix G Estimated renal survival at 3 years, 5 years of IgAN after excluding cohorts with enrollment periods longer than 10 years

Appendix H The 3-year, 5-year and 10-year median survival in developed countries and developing countries

Appendix A Literature Database Search Terms

| PubMed | (((((((((((((Berger's Disease[Title/Abstract]) OR (Bergers Disease[Title/Abstract])) OR (IGA Glomerulonephritis[Title/Abstract])) OR (IGA Nephropathy[Title/Abstract])) OR (Immunoglobulin A Nephropathy[Title/Abstract])) OR (Nephropathy Immunoglobulin A[Title/Abstract])) OR (Nephritis IGA Type[Title/Abstract])) OR (IGA Type Nephritis[Title/Abstract])) OR (Nephropathy IGA[Title/Abstract])) OR (Berger Disease[Title/Abstract])) OR (Iga Nephropathy 1[Title/Abstract])) OR (Nephropathy 1 Iga[Title/Abstract])) OR ("Glomerulonephritis IGA"[Mesh])) AND (((("Survival Analysis"[Mesh]) OR ("Prognosis"[Mesh])) OR ((Prognoses[Title/Abstract]) OR (Prognostic Factors[Title/Abstract]))) OR (((((((("kidney transplantation"[MeSH Terms]) OR ("renal dialysis"[MeSH Terms])) OR ("kidney failure chronic"[MeSH Terms])) OR ("treatment outcome"[MeSH Terms])) ) OR (ESRD[Title/Abstract])) OR (Chronic Renal Failure[Title/Abstract])) OR (End Stage Kidney Disease[Title/Abstract]))) AND (((Randomized Controlled Trial[Publication Type])) OR ((cohort [Title/Abstract]))) |
| --- | --- |
| Embase | #1 'prognosis'/exp  #2 'survival analysis'/exp  #3 'end stage renal disease'/exp  #4 'treatment outcome'/exp  #5 'kidney failure'/exp  #6 'berger disease':ab, ti  #7 ' iga glomerulonephritis':ab, ti  #8 'immunoglobulin a nephropathy'/exp  #9 #1 OR #2 OR #3 OR #4 OR #5  #10 #6 OR #7 OR #8  #11 #9 AND #10  #12 #11 AND('cohort analysis'/de OR 'randomized controlled trial'/de) |
| Cochrane Database | Glomerulonephritis, IGA |

Appendix B Study Quality.

| Cohort Study | Publication date | Representative of the exposed cohort | Selection of the non exposed cohort | Ascertainment of exposure | Outcome of interest was not present at start of study | Comparability | Assessment of outcome | Follow-up long enough | Adequate follow-up (≤20% attrition) | Total |
| --- | --- | --- | --- | --- | --- | --- | --- | --- | --- | --- |
| Hu Y | 2023 | 1 | 1 | 1 | 1 | 1 | 1 | 1 | 0 | 7 |
| Pitcher D | 2023 | 1 | 1 | 1 | 0 | 0 | 1 | 1 | 1 | 6 |
| Gadola L | 2023 | 1 | 1 | 1 | 1 | 1 | 1 | 1 | 0 | 7 |
| Zhu B | 2022 | 1 | 1 | 1 | 1 | 1 | 0 | 1 | 1 | 7 |
| Weng M | 2022 | 1 | 1 | 1 | 1 | 1 | 1 | 1 | 1 | 8 |
| Qin A | 2022 | 1 | 1 | 1 | 1 | 1 | 1 | 1 | 1 | 8 |
| Itami S | 2022 | 1 | 1 | 1 | 1 | 1 | 1 | 1 | 1 | 8 |
| Xu X | 2022 | 1 | 1 | 1 | 1 | 0 | 1 | 1 | 1 | 7 |
| Oh TR | 2022 | 1 | 1 | 1 | 1 | 1 | 1 | 1 | 0 | 7 |
| Haaskjold YL | 2022 | 1 | 1 | 1 | 1 | 1 | 1 | 1 | 1 | 8 |
| Yang X | 2021 | 1 | 1 | 1 | 1 | 1 | 1 | 1 | 1 | 8 |
| Varughese S | 2021 | 1 | 1 | 1 | 1 | 0 | 0 | 1 | 1 | 7 |
| Tan L | 2021 | 1 | 1 | 1 | 1 | 1 | 1 | 1 | 1 | 8 |
| Zhong ZX | 2021 | 1 | 1 | 1 | 1 | 1 | 1 | 1 | 1 | 8 |
| Wu L | 2021 | 1 | 1 | 1 | 1 | 1 | 1 | 1 | 1 | 8 |
| Wu J | 2021 | 1 | 1 | 1 | 1 | 1 | 1 | 1 | 1 | 8 |
| Wen D | 2021 | 0 | 1 | 1 | 1 | 0 | 1 | 1 | 1 | 6 |
| Wang S | 2021 | 1 | 1 | 1 | 1 | 1 | 1 | 1 | 1 | 8 |
| Wang M | 2021 | 1 | 1 | 1 | 1 | 1 | 1 | 1 | 1 | 8 |
| Tang X | 2021 | 1 | 1 | 1 | 1 | 0 | 1 | 1 | 1 | 7 |
| Tan L | 2021 | 1 | 1 | 1 | 1 | 1 | 1 | 1 | 1 | 8 |
| Sugiura N | 2021 | 1 | 1 | 1 | 1 | 1 | 1 | 1 | 1 | 8 |
| Schena FP | 2021 | 1 | 1 | 1 | 1 | 1 | 1 | 1 | 0 | 7 |
| Sato Y | 2021 | 1 | 1 | 1 | 1 | 1 | 1 | 1 | 1 | 8 |
| Oh TR | 2021 | 1 | 1 | 1 | 1 | 0 | 1 | 1 | 1 | 7 |
| Chang D | 2021 | 0 | 1 | 1 | 1 | 1 | 1 | 1 | 1 | 7 |
| Zhang J | 2021 | 1 | 1 | 1 | 1 | 2 | 1 | 1 | 0 | 8 |
| Worawichawong S | 2021 | 0 | 1 | 1 | 1 | 1 | 1 | 1 | 1 | 7 |
| Jiang Z | 2021 | 1 | 1 | 1 | 1 | 2 | 1 | 1 | 1 | 9 |
| Huang Z | 2021 | 1 | 1 | 1 | 1 | 1 | 1 | 1 | 1 | 8 |
| Zhu B | 2020 | 1 | 1 | 1 | 1 | 0 | 1 | 1 | 1 | 7 |
| Zhang Y | 2020 | 1 | 1 | 1 | 1 | 0 | 1 | 1 | 0 | 6 |
| Yang Y | 2020 | 1 | 1 | 1 | 1 | 1 | 1 | 1 | 1 | 8 |
| Xia M | 2020 | 1 | 1 | 1 | 1 | 0 | 1 | 1 | 0 | 6 |
| Wu H | 2020 | 1 | 1 | 1 | 1 | 0 | 1 | 1 | 1 | 7 |
| Wen Q | 2020 | 1 | 1 | 1 | 1 | 0 | 1 | 1 | 0 | 6 |
| Russo E | 2020 | 0 | 1 | 1 | 1 | 1 | 1 | 1 | 0 | 6 |
| Rauen T | 2020 | 0 | 1 | 1 | 1 | 1 | 1 | 1 | 1 | 7 |
| Qin A | 2020 | 1 | 1 | 1 | 1 | 0 | 1 | 1 | 1 | 7 |
| Peng S | 2020 | 1 | 1 | 1 | 1 | 2 | 1 | 0 | 1 | 8 |
| Pei G | 2020 | 1 | 1 | 1 | 1 | 2 | 1 | 1 | 1 | 9 |
| Neves PDMde M | 2020 | 0 | 1 | 1 | 1 | 1 | 1 | 1 | 0 | 6 |
| Neves PDMDM | 2020 | 0 | 1 | 1 | 1 | 0 | 1 | 1 | 1 | 6 |
| Moriyama T | 2020 | 1 | 1 | 1 | 1 | 1 | 1 | 1 | 1 | 8 |
| Ma F | 2020 | 0 | 1 | 1 | 1 | 2 | 1 | 0 | 1 | 7 |
| Ma F | 2020 | 0 | 1 | 1 | 1 | 1 | 1 | 1 | 1 | 7 |
| Luo R | 2020 | 1 | 1 | 1 | 1 | 0 | 1 | 1 | 0 | 6 |
| Li Q | 2020 | 1 | 1 | 1 | 1 | 1 | 1 | 1 | 1 | 8 |
| Kumon S | 2020 | 0 | 1 | 1 | 1 | 2 | 1 | 1 | 0 | 7 |
| Imai E | 2020 | 1 | 1 | 1 | 1 | 0 | 1 | 1 | 1 | 7 |
| Coppo R | 2020 | 1 | 1 | 1 | 1 | 0 | 1 | 1 | 0 | 6 |
| Chen T | 2020 | 1 | 1 | 1 | 1 | 1 | 1 | 1 | 1 | 8 |
| Chen CH | 2020 | 1 | 1 | 1 | 1 | 2 | 1 | 1 | 1 | 9 |
| Wang J | 2020 | 0 | 1 | 1 | 1 | 2 | 1 | 1 | 1 | 8 |
| Ai Z | 2020 | 1 | 1 | 1 | 1 | 0 | 1 | 1 | 1 | 7 |
| Tan M | 2019 | 1 | 1 | 1 | 1 | 1 | 1 | 1 | 0 | 7 |
| Peng W | 2019 | 1 | 1 | 1 | 1 | 0 | 1 | 1 | 1 | 7 |
| Obrișcă B | 2019 | 1 | 1 | 1 | 1 | 2 | 1 | 1 | 0 | 8 |
| Liu D | 2019 | 1 | 1 | 1 | 1 | 1 | 1 | 1 | 1 | 8 |
| Kaihan AB | 2019 | 0 | 1 | 1 | 1 | 2 | 1 | 1 | 1 | 8 |
| Jarrick S | 2019 | 1 | 1 | 1 | 1 | 1 | 1 | 1 | 1 | 8 |
| Hirano K | 2019 | 1 | 1 | 1 | 1 | 1 | 1 | 1 | 1 | 8 |
| Han X | 2019 | 1 | 1 | 1 | 1 | 0 | 1 | 1 | 1 | 7 |
| Duan SW | 2019 | 1 | 1 | 1 | 1 | 1 | 1 | 1 | 0 | 7 |
| Coppo R | 2019 | 0 | 1 | 1 | 1 | 0 | 1 | 1 | 0 | 5 |
| Chen T | 2019 | 1 | 1 | 1 | 1 | 1 | 1 | 1 | 1 | 8 |
| Chen P | 2019 | 1 | 1 | 1 | 1 | 1 | 1 | 1 | 1 | 8 |
| Bi TD | 2019 | 1 | 1 | 1 | 1 | 1 | 1 | 1 | 0 | 7 |
| Bellur SS | 2019 | 1 | 1 | 1 | 1 | 1 | 1 | 1 | 1 | 8 |
| Cai Q | 2019 | 1 | 1 | 1 | 1 | 0 | 1 | 1 | 1 | 7 |
| Ouyang Y | 2019 | 1 | 1 | 1 | 1 | 1 | 1 | 1 | 0 | 7 |
| Zhu L | 2018 | 1 | 1 | 1 | 1 | 0 | 1 | 1 | 1 | 7 |
| Zhu B | 2018 | 1 | 1 | 1 | 1 | 0 | 1 | 1 | 1 | 7 |
| Zhang X | 2018 | 1 | 1 | 1 | 1 | 1 | 1 | 1 | 1 | 8 |
| Zhang L | 2018 | 1 | 1 | 1 | 1 | 0 | 1 | 1 | 0 | 6 |
| Tanaka S | 2018 | 1 | 1 | 1 | 1 | 1 | 1 | 1 | 1 | 8 |
| Stangou M | 2018 | 1 | 1 | 1 | 1 | 2 | 1 | 1 | 0 | 8 |
| Sim JJ | 2018 | 1 | 1 | 1 | 1 | 0 | 1 | 1 | 0 | 6 |
| Shi M | 2018 | 1 | 1 | 1 | 1 | 0 | 1 | 1 | 1 | 7 |
| Park S | 2018 | 1 | 1 | 1 | 1 | 2 | 1 | 1 | 1 | 9 |
| Pan M | 2018 | 1 | 1 | 1 | 1 | 0 | 1 | 1 | 1 | 7 |
| Matsumoto K | 2018 | 1 | 1 | 1 | 1 | 1 | 1 | 1 | 1 | 8 |
| Kawai Y | 2018 | 1 | 1 | 1 | 1 | 0 | 1 | 1 | 1 | 7 |
| Deng W | 2018 | 1 | 1 | 1 | 1 | 1 | 1 | 1 | 0 | 7 |
| Chen S | 2018 | 1 | 1 | 1 | 1 | 1 | 1 | 1 | 0 | 7 |
| Liu Ll | 2018 | 1 | 1 | 1 | 1 | 1 | 1 | 1 | 0 | 7 |
| Zhu X | 2017 | 1 | 1 | 1 | 1 | 1 | 1 | 1 | 0 | 7 |
| Zhang W | 2017 | 1 | 1 | 1 | 1 | 1 | 1 | 1 | 1 | 8 |
| Zhang JJ | 2017 | 1 | 1 | 1 | 1 | 0 | 1 | 1 | 1 | 7 |
| Zhang J | 2017 | 0 | 1 | 1 | 1 | 1 | 1 | 1 | 1 | 7 |
| Yang X | 2017 | 1 | 1 | 1 | 1 | 2 | 1 | 1 | 1 | 9 |
| Yang M | 2017 | 1 | 1 | 1 | 1 | 1 | 1 | 1 | 1 | 8 |
| Wang W | 2017 | 1 | 1 | 1 | 1 | 1 | 1 | 1 | 1 | 8 |
| Sevillano AM | 2017 |  | 1 | 1 | 1 | 2 | 1 | 1 | 1 | 8 |
| Matsukuma Y | 2017 | 1 | 1 | 1 | 1 | 1 | 1 | 1 | 1 | 8 |
| Liu J | 2017 | 1 | 1 | 1 | 1 | 2 | 1 | 1 | 1 | 9 |
| Haas M | 2017 | 1 | 1 | 1 | 1 | 1 | 1 | 1 | 1 | 8 |
| Guo Wy | 2017 | 1 | 1 | 1 | 1 | 2 | 1 | 1 | 1 | 9 |
| Woo KT | 2016 | 0 | 1 | 1 | 1 | 2 | 1 | 1 | 1 | 8 |
| Tanaka S | 2016 | 1 | 1 | 1 | 1 | 0 | 1 | 1 | 1 | 7 |
| Sarcina C | 2016 | 1 | 1 | 1 | 1 | 1 | 1 | 1 | 1 | 8 |
| Ruggajo P | 2016 | 1 | 1 | 1 | 1 | 1 | 1 | 1 | 1 | 8 |
| Ouyang Y | 2016 | 1 | 1 | 1 | 1 | 0 | 1 | 1 | 1 | 7 |
| Kaneko Y | 2016 | 1 | 1 | 1 | 1 | 0 | 1 | 1 | 1 | 7 |
| Kamei K | 2016 | 0 | 1 | 1 | 1 | 0 | 1 | 1 | 1 | 6 |
| Hoshino J | 2016 | 1 | 1 | 1 | 1 | 0 | 1 | 1 | 1 | 7 |
| Chakera A | 2016 | 0 | 1 | 1 | 1 | 1 | 1 | 1 | 1 | 7 |
| Yuan Y | 2015 | 0 | 1 | 1 | 1 | 0 | 1 | 1 | 1 | 6 |
| Yuan Y | 2015 | 1 | 1 | 1 | 1 | 1 | 1 | 1 | 1 | 8 |
| Tanaka S | 2015 | 1 | 1 | 1 | 1 | 0 | 1 | 1 | 1 | 7 |
| Sato R | 2015 | 1 | 1 | 1 | 1 | 0 | 1 | 1 | 1 | 7 |
| Ruan Y | 2015 | 0 | 1 | 1 | 1 | 1 | 0 | 1 | 1 | 6 |
| Rhee H | 2015 | 0 | 1 | 1 | 1 | 0 | 1 | 1 | 1 | 6 |
| Moriyama T | 2015 | 1 | 1 | 1 | 1 | 2 | 1 | 1 | 0 | 8 |
| Liu M | 2015 | 0 | 1 | 1 | 1 | 1 | 1 | 1 | 1 | 7 |
| Cheungpasitporn W | 2015 | 1 | 1 | 1 | 1 | 1 | 1 | 1 | 1 | 8 |
| Arroyo A H | 2015 | 0 | 1 | 1 | 1 | 0 | 1 | 1 | 1 | 6 |
| Park KS | 2014 | 1 | 1 | 1 | 1 | 0 | 1 | 1 | 1 | 7 |
| Nam KH | 2014 | 1 | 1 | 1 | 1 |  | 1 | 1 | 1 | 7 |
| Moriyama T | 2014 | 1 | 1 | 1 | 1 | 1 | 1 | 1 | 1 | 8 |
| Maixnerova D | 2014 | 1 | 1 | 1 | 1 | 1 | 1 | 1 | 1 | 8 |
| Liu Y | 2014 | 1 | 1 | 1 | 1 | 2 | 1 | 1 | 1 | 9 |
| Li X | 2014 | 1 | 1 | 1 | 1 | 0 | 1 | 1 | 1 | 7 |
| Lee H | 2014 | 1 | 1 | 1 | 1 | 1 | 1 | 1 | 1 | 8 |
| Le W | 2014 | 1 | 1 | 1 | 1 | 0 | 1 | 1 | 1 | 7 |
| Kovács T | 2014 | 0 | 1 | 1 | 1 | 0 | 1 | 1 | 1 | 6 |
| Espinosa M | 2014 | 1 | 1 | 1 | 1 | 0 | 1 | 1 | 1 | 7 |
| Coppo R | 2014 | 1 | 1 | 1 | 1 | 1 | 1 | 1 | 1 | 8 |
| Zhang J | 2013 | 0 | 1 | 1 | 1 | 1 | 1 | 1 | 1 | 7 |
| Knoop T | 2013 | 1 | 1 | 1 | 1 | 0 | 1 | 1 | 1 | 7 |
| Barbour SJ | 2013 | 1 | 1 | 1 | 1 | 1 | 1 | 1 | 1 | 8 |
| Zhao N | 2012 | 1 | 1 | 1 | 1 | 1 | 1 | 1 | 1 | 8 |
| Shi Y | 2012 | 1 | 1 | 1 | 1 | 2 | 1 | 1 | 1 | 9 |
| Lundberg S | 2012 | 1 | 1 | 1 | 1 | 0 | 1 | 1 | 1 | 7 |
| Lee H | 2012 | 1 | 1 | 1 | 1 | 1 | 1 | 1 | 1 | 8 |
| Le WB | 2012 | 1 | 1 | 1 | 1 | 1 | 1 | 1 | 1 | 8 |
| Gutiérrez E | 2012 | 1 | 1 | 1 | 1 | 1 | 1 | 1 | 1 | 8 |
| Chou YH | 2012 | 1 | 1 | 1 | 1 | 0 | 1 | 1 | 1 | 7 |
| Bjørneklett R | 2012 | 1 | 1 | 1 | 1 | 0 | 1 | 1 | 1 | 7 |
| Katafuchi R | 2011 | 1 | 1 | 1 | 1 | 0 | 1 | 1 | 1 | 7 |
| Alamartine E | 2011 | 1 | 1 | 1 | 1 | 0 | 1 | 1 | 1 | 7 |
| Walsh M | 2010 | 1 | 1 | 1 | 1 | 1 | 1 | 1 | 1 | 8 |
| Chin H J | 2009 | 1 | 1 | 1 | 1 | 0 | 1 | 1 | 1 | 7 |
| Yata N | 2008 | 1 | 1 | 1 | 1 | 1 | 1 | 1 | 1 | 8 |
| Katafuchi R | 2008 | 1 | 1 | 1 | 1 | 0 | 1 | 1 | 1 | 7 |
| Berthoux F C | 2006 | 1 | 1 | 1 | 1 | 2 | 1 | 1 | 1 | 9 |
| Panzer U | 2005 | 1 | 1 | 1 | 1 | 1 | 1 | 1 | 1 | 8 |
| Nozawa R | 2005 | 1 | 1 | 1 | 1 | 1 | 1 | 1 | 1 | 8 |
| Descamps-Latscha B | 2004 | 1 | 1 | 1 | 1 | 1 | 1 | 1 | 1 | 8 |
| Geddes C C | 2003 | 1 | 1 | 1 | 1 | 0 | 1 | 1 | 0 | 6 |
| Li PKT | 2002 | 0 | 1 | 1 | 1 | 0 | 1 | 1 | 1 | 6 |
| Donadio JV | 1999 | 1 | 1 | 1 | 1 | 1 | 1 | 1 | 1 | 8 |
| Frimat L | 1997 | 1 | 1 | 1 | 1 | 1 | 1 | 1 | 1 | 8 |
| Frimat L | 1996 | 1 | 1 | 1 | 1 | 1 | 1 | 1 | 1 | 8 |
| Kang S W | 1995 | 1 | 1 | 1 | 1 | 1 | 1 | 1 | 1 | 8 |
| Woo K T | 1988 | 0 | 1 | 1 | 1 | 1 | 1 | 1 | 1 | 7 |

| Study（Randomized Clinical Trial） | Publication date | Random sequence genera  tion | Allocation concealment | Blinding of participants and personnel | Blinding of outcome assess  ment | Incomplete outcome data | Selective reporting | Other bias | Total |
| --- | --- | --- | --- | --- | --- | --- | --- | --- | --- |
| Lv J | 2022 | 1 | 0 | 1 | 1 | 1 | 1 | 0 | 5 |
| Lv J | 2017 | 1 | 1 | 1 | 1 | 1 | 1 | 0 | 6 |

Appendix C Characteristics of studies

| study | Publication date | Nationality | Sample size | Design | Male, | Age, years | Follow-up(months) | Reference |
| --- | --- | --- | --- | --- | --- | --- | --- | --- |
| Hu Y | 2023 | China | 222 | cohort study | 0.53 | 41 | 39 | 119 |
| Pitcher D | 2023 | United Kingdom | 2369 | cohort study | 0.71 | 41 | 70 | 153 |
| Gadola L | 2023 | Uruguay | 241 | cohort study | 0.64 | 32 | 120 | 154 |
| Xu X | 2022 | China | 105 | cohort study | 0.49 | 36 | 37 | 147 |
| Oh T R | 2022 | Korea | 2945 | cohort study | 0.48 | 40 | 72 | 148 |
| Lv J | 2022 | China | 503 | Randomized Clinical Trial | 0.61 | 38 | 50 | 149 |
| Haaskjold YL | 2022 | Norway | 306 | cohort study | 0.76 | 37 | 198 | 150 |
| Zhu B | 2022 | China | 1420 | cohort study | 0.42 | 33 | 84 | 155 |
| Weng M | 2022 | China | 152 | cohort study | 0.76 | 35 | 58 | 156 |
| Qin A | 2022 | China | 1210 | cohort study | 0.44 | 32 | 56 | 157 |
| Itami S | 2022 | Japan | 1147 | cohort study | 0.42 | 31 | 120 | 158 |
| Varughese S | 2022 | India | 195 | cohort study | 0.7 | 36 | 36 | 125 |
| Yang X | 2021 | China | 208 | cohort study | 0.58 | 36 | 42 | 151 |
| Tan L | 2021 | China | 1239 | cohort study | 0.43 | 33 | 50 | 152 |
| Zhong ZX | 2021 | China | 449 | cohort study | 0.48 | 35 | 44 | 1 |
| Wu L | 2021 | China | 337 | cohort study | 0.43 | 30 | 64 | 3 |
| Wu J | 2021 | China | 136 | cohort study | 0.49 | 36 | 105 | 4 |
| Wen D | 2021 | China | 1096 | cohort study | 0.43 | 34 | 41 | 6 |
| Wang S | 2021 | China | 966 | cohort study | 0.46 | 33 | 58 | 7 |
| Wang M | 2021 | China | 1313 | cohort study | 0.51 | 35 | 44 | 8 |
| Tang X | 2021 | China | 1570 | cohort study | 0.27 | 32 | 49 | 9 |
| Tan L | 2021 | China | 1071 | cohort study | 0.43 | 34 | 42 | 10 |
| Sugiura N | 2021 | Japan | 678 | cohort study | 0.40 | 31 | 240 | 11 |
| Schena FP | 2021 | Italy | 948 | cohort study | 0.72 | 41 | 89 | 12 |
| Sato Y | 2021 | Japan | 467 | cohort study | 0.40 | 37 | 107 | 13 |
| Oh TR | 2021 | Korea | 4326 | cohort study | 0.49 | 39 | 73 | 14 |
| Chang D | 2021 | China | 330 | cohort study | 0.42 | 34 | 47 | 17 |
| Zhang J | 2021 | China | 296 | cohort study | 0.64 | 36 | 36 | 2 |
| Worawichawong S | 2021 | Thailand | 120 | cohort study | 0.47 | 37 | 52 | 5 |
| Jiang Z | 2021 | China | 1492 | cohort study | 0.55 | 33 | 60 | 15 |
| Huang Z | 2021 | China | 200 | cohort study | 0.41 | 43 | 40 | 16 |
| Zhu B | 2020 | China | 1828 | cohort study | - | 34 | 84 | 19 |
| Zhang Y | 2020 | China | 397 | cohort study | 0.55 | 35 | 75 | 20 |
| Yang Y | 2020 | China | 642 | cohort study | 0.44 | 33 | 43 | 21 |
| Xia M | 2020 | China | 291 | cohort study | 0.45 | 32 | 41 | 22 |
| Wu H | 2020 | China | 1243 | cohort study | 0.68 | 14 | 87 | 23 |
| Wen Q | 2020 | China | 981 | cohort study | 0.42 | 32 | 48 | 24 |
| Russo E | 2020 | Italy | 145 | cohort study | 0.71 | 47 | 68 | 26 |
| Rauen T | 2020 | Germany | 149 | cohort study | 0.76 | 46 | 89 | 27 |
| Qin A | 2020 | China | 715 | cohort study | 0.47 | 33 | 45 | 28 |
| Peng S | 2020 | China | 742 | cohort study | 0.56 | 36 | 31 | 29 |
| Pei G | 2020 | China | 295 | cohort study | 0.36 | 34 | 49 | 30 |
| Neves PM | 2020 | Brazil | 118 | cohort study | 0.45 | 33 | 65 | 31 |
| Neves PDMDM | 2020 | Brazil | 111 | cohort study | 0.41 | 32 | 64 | 32 |
| Moriyama T | 2020 | Japan | 871 | cohort study | 0.41 | 31 | 96 | 33 |
| Ma F | 2020 | China | 132 | cohort study | 0.76 | 36 | 33 | 34 |
| Ma F | 2020 | China | 338 | cohort study | 0.60 | 32 | 50 | 35 |
| Luo R | 2020 | China | 230 | cohort study | 0.40 | 34 | 41 | 36 |
| Li Q | 2020 | China | 1151 | cohort study | 0.50 | 34 | 45 | 37 |
| Kumon S | 2020 | Japan | 159 | cohort study | 0.38 | 42 | 120 | 38 |
| Imai E | 2020 | Japan | 267 | cohort study | 0.60 | 38 | 166 | 39 |
| Coppo R | 2020 | United Kingdom | 1130 | cohort study | 0.73 | 35 | 84 | 40 |
| Chen T | 2020 | China | 4047 | cohort study | 0.54 | 34 | 156 | 41 |
| Chen CH | 2020 | Italy | 388 | cohort study | 0.55 | 41 | 86 | 42 |
| Wang J | 2020 | China | 172 | cohort study | 0.44 | 36 | 39 | 25 |
| Ai Z | 2020 | China | 921 | cohort study | 0.42 | 32 | 48 | 43 |
| Tan M | 2019 | China | 1069 | cohort study | 0.12 | 32 | 60 | 44 |
| Peng W | 2019 | China | 1328 | cohort study | 0.46 | 34 | 46 | 45 |
| Obrișcă B | 2019 | Romania | 248 | cohort study | 0.71 | 43 | 32 | 46 |
| Liu D | 2019 | China | 455 | cohort study | 0.44 | 32 | 42 | 47 |
| Kaihan AB | 2019 | Japan | 104 | cohort study | 0.44 | 35 | 75 | 48 |
| Jarrick S | 2019 | Sweden | 3622 | cohort study | 0.70 | 40 | 163 | 49 |
| Hirano K | 2019 | Japan | 1065 | cohort study | 0.50 | 35 | 120 | 50 |
| Han X | 2019 | China | 1165 | cohort study | 0.45 | 34 | 44 | 51 |
| Duan SW | 2019 | China | 412 | cohort study | 0.54 | 35 | 81 | 52 |
| Coppo R | 2019 | Italy | 174 | cohort study | 0.72 | 13 | 53 | 53 |
| Chen T | 2019 | China | 2047 | cohort study | 0.51 | 35 | 84 | 54 |
| Chen P | 2019 | China | 1210 | cohort study | 0.51 | 35 | 43 | 55 |
| Bi TD | 2019 | China | 1157 | cohort study | 0.50 | 37 | 80 | 57 |
| Bellur SS | 2019 | Europe | 503 | cohort study | - | - | 180 | 58 |
| Cai Q | 2019 | China | 944 | cohort study | 0.51 | 36 | 50 | 56 |
| Ouyang Y | 2019 | China | 1007 | cohort study | 1.26 | 36 | 41 | 59 |
| Zhu L | 2018 | China | 1126 | cohort study | 0.51 | 33 | 44 | 60 |
| Zhu B | 2018 | China | 1965 | cohort study | 0.43 | 33 | 84 | 61 |
| Zhang X | 2018 | China | 1152 | cohort study | 0.49 | 35 | 169 | 62 |
| Zhang L | 2018 | China | 988 | cohort study | 0.42 | 33 | 49 | 63 |
| Tanaka S | 2018 | Japan | 1255 | cohort study | 0.58 | 35 | 52 | 64 |
| Stangou M | 2018 | Greece | 457 | cohort study | 0.66 | 41 | 64 | 65 |
| Sim JJ | 2018 | America | 269 | cohort study | 4.13 | 44 | 54 | 66 |
| Shi M | 2018 | China | 517 | cohort study | 0.51 | 38 | 50 | 67 |
| Park S | 2018 | Korea | 118 | cohort study | 0.00 | 27 | 86 | 68 |
| Pan M | 2018 | China | 712 | cohort study | 0.43 | 37 | 41 | 69 |
| Matsumoto K | 2018 | Japan | 227 | cohort study | 0.39 | 34 | 92 | 70 |
| Kawai Y | 2018 | Japan | 1352 | cohort study | 0.45 | 35 | 61 | 72 |
| Deng W | 2018 | China | 988 | cohort study | 0.42 | 32 | 49 | 73 |
| Chen S | 2018 | China | 311 | cohort study | 0.38 | 36 | 38 | 74 |
| Liu Ll | 2018 | China | 869 | cohort study | 0.51 | 34 | 44 | 71 |
| Zhu X | 2017 | China | 742 | cohort study | 0.44 | 33 | 66 | 75 |
| Zhang W | 2017 | China | 538 | cohort study | 0.44 | 32 | 51 | 76 |
| Zhang JJ | 2017 | China | 390 | cohort study | 0.68 | - | 42 | 77 |
| Zhang J | 2017 | China | 244 | cohort study | 0.48 | 39 | 82 | 78 |
| Yang X | 2017 | China | 496 | cohort study | 0.66 | 39 | 34 | 79 |
| Yang M | 2017 | China | 919 | cohort study | 0.49 | 36 | 57 | 80 |
| Wang W | 2017 | China | 521 | cohort study | 0.44 | 34 | 44 | 81 |
| Sevillano AM | 2017 | Spain | 112 | cohort study | 0.71 | 42 | 168 | 82 |
| Matsukuma Y | 2017 | Japan | 1218 | cohort study | 0.45 | 35 | 180 | 83 |
| Lv J | 2017 | China | 262 | Randomized Clinical Trial | 0.63 | 39 | 60 | 84 |
| Liu J | 2017 | China | 349 | cohort study | 0.90 | 35 | 84 | 85 |
| Haas M | 2017 | America | 3096 | cohort study | 0.58 | 35 | 56 | 86 |
| Guo Wy | 2017 | China | 749 | cohort study | 0.48 | 35 | 38 | 87 |
| Woo KT | 2016 | Singapore | 102 | cohort study | 0.71 | 24 | 180 | 88 |
| Tanaka S | 2016 | Japan | 1273 | cohort study | 0.46 | 36 | 61 | 89 |
| Sarcina C | 2016 | Italy | 325 | cohort study | 0.74 | 38 | 67 | 90 |
| Ruggajo P | 2016 | Norway | 471 | cohort study | 0.70 | 24 | 120 | 91 |
| Ouyang Y | 2016 | China | 930 | cohort study | 0.49 | 38 | 47 | 92 |
| Kaneko Y | 2016 | Japan | 314 | cohort study | 0.48 | 35 | 106 | 93 |
| Kamei K | 2016 | Japan | 100 | cohort study | 0.50 | 12 | 142 | 94 |
| Hoshino J | 2016 | Japan | 1127 | cohort study | 0.57 | 44 | 240 | 95 |
| Chakera A | 2016 | United Kingdom | 147 | cohort study | 0.76 | 40 | 82 | 96 |
| Yuan Y | 2015 | China | 200 | cohort study | 0.44 | 37 | 63 | 97 |
| Yuan Y | 2015 | China | 657 | cohort study | 0.58 | 38 | 66 | 98 |
| Tanaka S | 2015 | Japan | 694 | cohort study | 0.48 | 36 | 59 | 99 |
| Sato R | 2015 | Japan | 198 | cohort study | 0.48 | 44 | 144 | 100 |
| Ruan Y | 2015 | China | 206 | cohort study | 0.42 | 33 | 28 | 101 |
| Rhee H | 2015 | Korea | 121 | cohort study | 0.60 | 33 | 41 | 102 |
| Moriyama T | 2015 | Japan | 103 | cohort study | 0.52 | - | 360 | 103 |
| Liu M | 2015 | China | 351 | cohort study | 0.52 | 34 | 52 | 104 |
| Cheungpasitporn W | 2015 | America | 207 | cohort study | 0.69 | 49 | 51 | 105 |
| Arroyo A H | 2015 | Spain | 126 | cohort study | 0.64 | 34 | 47 | 106 |
| Park KS | 2014 | Korea | 500 | cohort study | 0.43 | 37 | 68 | 107 |
| Nam KH | 2014 | Korea | 500 | cohort study | 0.43 | 37 | 65 | 108 |
| Moriyama T | 2014 | Japan | 1012 | cohort study | 0.41 | 33 | 160 | 109 |
| Maixnerova D | 2014 | Czech Republic | 520 | cohort study | 0.69 | 39 | 72 | 110 |
| Liu Y | 2014 | China | 124 | cohort study | 0.00 | 27 | 46 | 111 |
| Li X | 2014 | China | 703 | cohort study | 0.51 | 34 | 45 | 112 |
| Lee H | 2014 | Korea | 153 | cohort study | 0.39 | 26 | 95 | 112 |
| Le W | 2014 | China | 1155 | cohort study | 0.47 | - | 95 | 114 |
| Kovács T | 2014 | Hungary | 264 | cohort study | 0.73 | 34 | 130 | 115 |
| Espinosa M | 2014 | Spain | 283 | cohort study | 0.73 | 39 | 240 | 116 |
| Coppo R | 2014 | 13 European countries | 1147 | cohort study | 0.73 | 36 | 56 | 117 |
| Zhang J | 2013 | China | 217 | cohort study | 0.41 | 30 | 36 | 118 |
| Knoop T | 2013 | Norway | 633 | cohort study | 0.74 | 39 | 142 | 120 |
| Barbour SJ | 2013 | Asia | 669 | cohort study | 0.62 | 40 | 46 | 121 |
| Zhao N | 2012 | China | 275 | cohort study | 0.53 | 33 | 47 | 122 |
| Shi Y | 2012 | America | 353 | cohort study | 0.39 | 35 | 60 | 123 |
| Lundberg S | 2012 | Sweden | 180 | cohort study | 0.71 | 41 | 55 | 124 |
| Lee H | 2012 | Korea | 1364 | cohort study | 0.50 | 33 | 360 | 126 |
| Le WB | 2012 | China | 1155 | cohort study | 0.50 | 34 | 240 | 126 |
| Gutiérrez E | 2012 | Spain | 141 | cohort study | 0.64 | 24 | 108 | 128 |
| Chou YH | 2012 | Taiwan | 580 | cohort study | 0.58 | 44 | 71 | 129 |
| Bjørneklett R | 2012 | Norway | 633 | cohort study | 0.74 | 39 | 124 | 130 |
| Katafuchi R | 2011 | Japan | 702 | cohort study | 0.42 | 30 | 62 | 131 |
| Alamartine E | 2011 | France | 183 | cohort study | 0.75 | 43 | 77 | 132 |
| Walsh M | 2010 | Canada | 137 | cohort study | 0.64 | 39 | 70 | 133 |
| Chin H J | 2009 | Korea | 1458 | cohort study | 0.56 | 37 | 45 | 134 |
| Yata N | 2008 | Japan | 500 | cohort study | 0.56 | 11 | 71 | 135 |
| Katafuchi R | 2008 | Japan | 702 | cohort study | 0.42 | 33 | 62 | 136 |
| Berthoux F C | 2006 | France | 318 | cohort study | 0.69 | 35 | 148 | 137 |
| Panzer U | 2005 | Germany | 228 | cohort study | 0.63 | 39 | 48 | 138 |
| Nozawa R | 2005 | Japan | 181 | cohort study | 0.59 | 12 | 88 | 139 |
| Descamps-Latscha B | 2004 | France | 120 | cohort study | 0.76 | 40 | 60 | 140 |
| Geddes C C | 2003 | United Kingdom, Finland, Australia and Canada | 711 | cohort study | 0.66 | 36 | 60 | 18 |
| Li PKT | 2002 | China | 167 | cohort study | 0.40 | 33 | 89 | 141 |
| Donadio JV | 1999 | America | 106 | cohort study | 0.75 | - | 77 | 142 |
| Frimat L | 1997 | France | 210 | cohort study | 0.82 | 36 | 67 | 143 |
| Frimat L | 1996 | France | 129 | cohort study | 0.81 | 38 | 41 | 144 |
| Kang S W | 1995 | Korea | 122 | cohort study | 0.61 | 28 | 43 | 145 |
| Woo K T | 1988 | Singapore | 151 | cohort study | 0.73 | 26 | 73 | 146 |

Appendix D List of Original Studies Included in the Meta-analysis

| 1. Zhong ZX, Y Tang, JX Tan, et al. Corticosteroids could improve the renal outcome of IgA nephropathy with moderate proteinuria. International Urology and Nephrology, 2021. 53(1): p. 121-127. |
| --- |
| 2. Zhang J, Y Wang, Z Liu, et al. Overlapping obesity-related glomerulopathy and immunoglobulin A nephropathy: clinical and pathologic characteristics and prognosis. Clinical and Experimental Nephrology, 2021. 25(8): p. 865-874. |
| 3. Wu L, D Liu, M Xia, et al. Immunofluorescence deposits in the mesangial area and glomerular capillary loops did not affect the prognosis of immunoglobulin a nephropathy except C1q: a single-center retrospective study. BMC Nephrology, 2021. 22(1). |
| 4. Wu J, Z Hu, Y Wang, et al. Severe glomerular C3 deposition indicates severe renal lesions and a poor prognosis in patients with immunoglobulin A nephropathy. Histopathology, 2021. 78(6): p. 882-895. |
| 5. Worawichawong S, S Plumworasawat, W Liwlompaisan, et al. Distribution pattern of mesangial C4d deposits as predictor of kidney failure in IgA nephropathy. PLoS ONE, 2021. 16(6 June). |
| 6. Wen D, Y Tang, L Tan, et al. Sex disparities in IgA nephropathy: a retrospective study in Chinese patients. International Urology and Nephrology, 2021. 53(2): p. 315-323. |
| 7. Wang S, L Dong, G Pei, et al. High Neutrophil-To-Lymphocyte Ratio Is an Independent Risk Factor for End Stage Renal Diseases in IgA Nephropathy. Frontiers in Immunology, 2021. 12. |
| 8. Wang M, J Lv, P Chen, et al. Associations of ABO blood type and galactose-deficient immunoglobulin A1 with adverse outcomes in patients with IgA nephropathy. Nephrology, dialysis, transplantation : official publication of the European Dialysis and Transplant Association - European Renal Association, 2021. 36(2): p. 288-294. |
| 9. Tang X, Q Wen, Q Zhou, et al. Clinicopathological characteristics and prognosis of patients with IgA nephropathy and renal vasculitic lesions. BMC Nephrology, 2021. 22(1). |
| 10. Tan L, Y Tang, G Pei, et al. A multicenter, prospective, observational study to determine association of mesangial C1q deposition with renal outcomes in IgA nephropathy. Scientific reports, 2021. 11(1): p. 5467. |
| 11. Sugiura N, T Moriyama, Y Miyabe, et al. Severity of arterial and/or arteriolar sclerosis in IgA nephropathy and the effects of renin–angiotensin system inhibitors on its prognosis. Journal of Pathology: Clinical Research, 2021. 7(6): p. 616-623. |
| 12. Schena FP, VW Anelli, J Trotta, et al. Development and testing of an artificial intelligence tool for predicting end-stage kidney disease in patients with immunoglobulin A nephropathy. Kidney International, 2021. 99(5): p. 1179-1188. |
| 13. Sato Y, H Tsukaguchi, K Higasa, et al. Positive renal familial history in IgA nephropathy is associated with worse renal outcomes: a single-center longitudinal study. BMC Nephrology, 2021. 22(1). |
| 14. Oh TR, SH Song, HS Choi, et al. The association between serum hemoglobin and renal prognosis of iga nephropathy. Journal of Clinical Medicine, 2021. 10(2): p. 1-11. |
| 15. Jiang Z, J Tan, S Wang, et al. Lower serum bilirubin is associated with poor renal outcome in iga nephropathy patients. International Journal of Medical Sciences, 2021. 18(13): p. 2964-2970. |
| 16. Huang Z, B Chen, Y Zhou, et al. Clinicopathological and prognostic study of IgA-dominant postinfectious glomerulonephritis. BMC Nephrology, 2021. 22(1). |
| 17. Chang D, Y Cheng, R Luo, et al. The prognostic value of platelet-to-lymphocyte ratio on the long-term renal survival in patients with IgA nephropathy. International Urology and Nephrology, 2021. 53(3): p. 523-530. |
| 18. Geddes CC, V Rauta, C Gronhagen-Riska, et al. A tricontinental view of IgA nephropathy. Nephrology Dialysis Transplantation, 2003. 18(8): p. 1541-1548. |
| 19. Zhu B, WH Liu, DR Yu, et al. The Association of Low Hemoglobin Levels with IgA Nephropathy Progression: A Two-Center Cohort Study of 1,828 Cases. American Journal of Nephrology, 2020. 51(8): p. 624-634. |
| 20. Zhang Y, SW Duan, P Chen, et al. Relationship between serum C3/C4 ratio and prognosis of immunoglobulin A nephropathy based on propensity score matching. Chin Med J (Engl), 2020. 133(6): p. 631-7. |
| 21. Yang Y, X Tang, Y Yang, et al. Glomerular C4 deposition and glomerulosclerosis predict worse renal outcomes in Chinese patients with IgA nephropathy. Renal Failure, 2020. 42(1): p. 629-637. |
| 22. Xia M, D Liu, L Peng, et al. Coagulation parameters are associated with the prognosis of immunoglobulin a nephropathy: a retrospective study. BMC Nephrology, 2020. 21(1). |
| 23. Wu H, X Fang, Z Xia, et al. Long-term renal survival and undetected risk factors of IgA nephropathy in Chinese children—a retrospective 1243 cases analysis from single centre experience. Journal of Nephrology, 2020. 33(6): p. 1263-1273. |
| 24. Wen Q, R Rong, Q Zhou, et al. Clinical, pathological characteristics and outcomes of immunoglobulin A nephropathy patients with different ages. Nephrology, 2020. 25(12): p. 906-912. |
| 25. Wang J, L He, W Yan, et al. The role of hypertriglyceridemia and treatment patterns in the progression of IgA nephropathy with a high proportion of global glomerulosclerosis. International Urology and Nephrology, 2020. 52(2): p. 325-335. |
| 26. Russo E, D Verzola, G Salvidio, et al. Long-term blood pressure behavior and progression to end-stage renal disease in patients with immunoglobulin A nephropathy: A single-center observational study in Italy. Journal of Hypertension, 2020. 38(5): p. 925-935. |
| 27. Rauen T, S Wied, C Fitzner, et al. After ten years of follow-up, no difference between supportive care plus immunosuppression and supportive care alone in IgA nephropathy. Kidney international, 2020. 98(4): p. 1044‐1052. |
| 28. Qin A, G Pei, Y Tang, et al. Corticosteroids Improve Renal Survival: A Retrospective Analysis From Chinese Patients With Early-Stage IgA Nephropathy. Frontiers in Medicine, 2020. 7. |
| 29. Peng S, W Lu, X Jiang, et al. IgG deposits in the mesangium and capillary loops predict poor renal outcome in patients with IgA nephropathy: a single-center retrospective study. Renal Failure, 2020. 42(1): p. 902-911. |
| 30. Pei G, J Tan, Y Tang, et al. Corticosteroids or immunosuppressants were not superior to supportive care in IgA nephropathy patients with mild proteinuria. Medicine (Baltimore), 2020. 99(24). |
| 31. Neves PM, RA Souza, FM Torres, et al. Evidences of histologic thrombotic microangiopathy and the impact in renal outcomes of patients with IgA nephropathy. PLoS One, 2020. 15(11). |
| 32. Neves PDMDM, RBB Pinheiro, CB Dias, et al. Renal Outcomes in Brazilian Patients with Immunoglobulin A Nephropathy and Cellular Crescentic Lesions. Kidney and Blood Pressure Research, 2020. 45(3): p. 431-441. |
| 33. Moriyama T, K Karasawa, Y Miyabe, et al. Validation of the revised Oxford classification for IgA nephropathy considering treatment with corticosteroids/immunosuppressors. Sci Rep, 2020. 10. |
| 34. Ma F, X Yang, M Zhou, et al. Treatment for IgA nephropathy with stage 3 or 4 chronic kidney disease: low-dose corticosteroids combined with oral cyclophosphamide. Journal of Nephrology, 2020. 33(6): p. 1241-1250. |
| 35. Ma F, L Liu, R Dong, et al. Renal survival and risk factors in IgA nephropathy with crescents. International Urology and Nephrology, 2020. 52(8): p. 1507-1516. |
| 36. Luo R, Y Yang, YC Cheng, et al. Plasma chemokine CXC motif-ligand 16 as a predictor of renal prognosis in immunoglobulin A nephropathy. Annals of Translational Medicine, 2020. 8(6). |
| 37. Li Q, P Chen, S Shi, et al. Neutrophil-to-lymphocyte ratio as an independent inflammatory indicator of poor prognosis in IgA nephropathy. International Immunopharmacology, 2020. 87. |
| 38. Kumon S, T Moriyama, T Kamiyama, et al. The impact of tonsillectomy combined with steroid pulse therapy in patients with advanced IgA nephropathy and impaired renal function. Clinical and Experimental Nephrology, 2020. 24(4): p. 295-306. |
| 39. Imai E, J Usui, S Kaneko, et al. The precise long-term outcomes of adult IgA nephropathy by mail questionnaires: Better renal survival compared to earlier cohort studies. PLoS One, 2020. 15(5). |
| 40. Coppo R, G D'Arrigo, G Tripepi, et al. Is there long-term value of pathology scoring in immunoglobulin A nephropathy? A validation study of the Oxford Classification for IgA Nephropathy (VALIGA) update. Nephrology Dialysis Transplantation, 2020. 35(6): p. 1002-1009. |
| 41. Chen T, E Xia, T Chen, et al. Identification and external validation of IgA nephropathy patients benefiting from immunosuppression therapy. EBioMedicine, 2020. 52. |
| 42. Chen CH, MJ Wu, MC Wen, et al. Crescents formations are independently associated with higher mortality in biopsy-confirmed immunoglobulin A nephropathy. PLoS One, 2020. 15(7). |
| 43. Ai Z, Q Zhou, F Huang, et al. Long-term renal outcomes of IgA nephropathy presenting with different levels of proteinuria. Clinical Nephrology, 2020. 94(6): p. 290-296. |
| 44. Tan M, J Fang, Q Xu, et al. Outcomes of normotensive IgA nephropathy patients with mild proteinuria who have impaired renal function. Ren Fail, 2019. 41(1): p. 875-82. |
| 45. Peng W, Y Tang, L Tan, et al. Crescents and global glomerulosclerosis in Chinese IgA nephropathy patients: A five-year follow-up. Kidney and Blood Pressure Research, 2019. 44(1): p. 103-112. |
| 46. Obrișcă B, G Ștefan, M Gherghiceanu, et al. “Associated” or “Secondary” IgA nephropathy? An outcome analysis. PLoS One, 2019. 14(8). |
| 47. Liu D, J You, Y Liu, et al. Serum immunoglobulin G provides early risk prediction in immunoglobulin A nephropathy. International Immunopharmacology, 2019. 66: p. 13-18. |
| 48. Kaihan AB, Y Yasuda, T Imaizumi, et al. Clinical impact of endocapillary proliferation with modified cutoff points in IgA nephropathy patients. PLoS One, 2019. 14(3). |
| 49. Jarrick S, S Lundberg, A Welander, et al. Mortality in IgA Nephropathy: A Nationwide Population-Based Cohort Study. J Am Soc Nephrol, 2019. 30(5): p. 866-76. |
| 50. Hirano K, K Matsuzaki, T Yasuda, et al. Association Between Tonsillectomy and Outcomes in Patients With Immunoglobulin A Nephropathy. JAMA Netw Open, 2019. 2(5). |
| 51. Han X, Y Xiao, Y Tang, et al. Clinical and pathological features of immunoglobulin A nephropathy patients with nephrotic syndrome. Clinical and Experimental Medicine, 2019. 19(4): p. 479-486. |
| 52. Duan SW, Y Mei, J Liu, et al. Predictive Capabilities of Three Widely Used Pathology Classification Systems and a Simplified Classification (Beijing Classification) in Primary IgA Nephropathy. Kidney and Blood Pressure Research, 2019. 44(5): p. 928-941. |
| 53. Coppo R. Pediatric IgA Nephropathy in Europe. Kidney Diseases, 2019. 5(3): p. 182-188. |
| 54. Chen T, X Li, Y Li, et al. Prediction and Risk Stratification of Kidney Outcomes in IgA Nephropathy. American Journal of Kidney Diseases, 2019. 74(3): p. 300-309. |
| 55. Chen P, G Yu, X Zhang, et al. Plasma galactose-deficient IgA1 and C3 and CKD progression in IgA nephropathy. Clinical Journal of the American Society of Nephrology, 2019. 14(10): p. 1458-1465. |
| 56. Cai Q, S Shi, S Wang, et al. Microangiopathic Lesions in IgA Nephropathy: A Cohort Study. American Journal of Kidney Diseases, 2019. 74(5): p. 629-639. |
| 57. Bi TD, JN Zheng, JX Zhang, et al. Serum complement C4 is an important prognostic factor for IgA nephropathy: A retrospective study. BMC Nephrology, 2019. 20(1). |
| 58. Bellur SS, ISD Roberts, S Troyanov, et al. Reproducibility of the Oxford classification of immunoglobulin A nephropathy, impact of biopsy scoring on treatment allocation and clinical relevance of disagreements: Evidence from the VALidation of IGA study cohort. Nephrology Dialysis Transplantation, 2019. 34(10): p. 1681-1690. |
| 59. Ouyang Y, L Zhu, M Shi, et al. A Rare Genetic Defect of MBL2 Increased the Risk for Progression of IgA Nephropathy. Frontiers in immunology, 2019. 10: p. 537. |
| 60. Zhu L, WY Guo, SF Shi, et al. Circulating complement factor H–related protein 5 levels contribute to development and progression of IgA nephropathy. Kidney International, 2018. 94(1): p. 150-158. |
| 61. Zhu B, DR Yu, JC Lv, et al. Uric Acid as a Predictor of Immunoglobulin A Nephropathy Progression: A Cohort Study of 1965 Cases. American Journal of Nephrology, 2018. 48(2): p. 127-136. |
| 62. Zhang X, S Shi, Y Ouyang, et al. A validation study of crescents in predicting ESRD in patients with IgA nephropathy. J Transl Med, 2018. 16. |
| 63. Zhang L, X Zhuang, and X Liao. A proposed Oxford classification-based clinicopathological nomogram for predicting short-term renal outcomes in IgA nephropathy after acute kidney injury. European Journal of Internal Medicine, 2018. 52: p. 60-66. |
| 64. Tanaka S, T Ninomiya, R Katafuchi, et al. Secular trends in the incidence of end-stage renal disease and its risk factors in Japanese patients with immunoglobulin A nephropathy. Nephrology Dialysis Transplantation, 2018. 33(6): p. 963-971. |
| 65. Stangou M, M Papasotiriou, D Xydakis, et al. IgA nephropathy in Greece: Data from the registry of the hellenic society of nephrology. Clinical Kidney Journal, 2018. 11(1): p. 38-45. |
| 66. Sim JJ, SK Bhandari, M Batech, et al. End-Stage Renal Disease and Mortality Outcomes Across Different Glomerulonephropathies in a Large Diverse US Population. Mayo Clinic Proceedings, 2018. 93(2): p. 167-178. |
| 67. Shi M, Y Ouyang, M Yang, et al. IgA nephropathy susceptibility loci and disease progression. Clinical Journal of the American Society of Nephrology, 2018. 13(9): p. 1330-1338. |
| 68. Park S, KD Yoo, JS Park, et al. Pregnancy in women with immunoglobulin A nephropathy: Are obstetrical complications associated with renal prognosis? Nephrology Dialysis Transplantation, 2018. 33(3): p. 459-465. |
| 69. Pan M, QX Zhou, SB Zheng, et al. Serum C3/C4 ratio is a novel predictor of renal prognosis in patients with IgA nephropathy: a retrospective study. Immunologic Research, 2018. 66(3): p. 381-391. |
| 70. Matsumoto K, Y Ikeda, S Yamaguchi, et al. Long-term outcomes of tonsillectomy for IgA nephropathy patients: A retrospective cohort study, two-centre analysis with the inverse probability therapy weighting method. Nephrology, 2018. 23(9): p. 846-854. |
| 71. Liu L, L Zhu, J Zheng, et al. Development and assessment of a predictive nomogram for the progression of IgA nephropathy. Sci Rep, 2018. 8. |
| 72. Kawai Y, K Masutani, K Torisu, et al. Association between serum albumin level and incidence of end-stage renal disease in patients with Immunoglobulin A nephropathy: A possible role of albumin as an antioxidant agent. PLoS One, 2018. 13(5). |
| 73. Deng W, X Tan, Q Zhou, et al. Gender-related differences in clinicopathological characteristics and renal outcomes of Chinese patients with IgA nephropathy. BMC Nephrol, 2018. 19. |
| 74. Chen S, Q Yin, S Ren, et al. A comparison of the effectiveness of cyclophosphamide, leflunomide, corticosteroids, or conservative management alone in patients with IgA nephropathy: a retrospective observational study. Sci Rep, 2018. 8. |
| 75. Zhu X, H Li, Y Liu, et al. Tubular atrophy/interstitial fibrosis scores of Oxford classification combinded with proteinuria level at biopsy provides earlier risk prediction in lgA nephropathy. Sci Rep, 2017. 7. |
| 76. Zhang W, Q Zhou, L Hong, et al. Clinical outcomes of IgA nephropathy patients with different proportions of crescents. Medicine (Baltimore), 2017. 96(11). |
| 77. Zhang JJ, GZ Yu, ZH Zheng, et al. Dividing CKD stage 3 into G3a and G3b could better predict the prognosis of IgA nephropathy. PLoS One, 2017. 12(4). |
| 78. Zhang J, P Ren, Y Wang, et al. Serum Matrix Metalloproteinase-7 Level is Associated with Fibrosis and Renal Survival in Patients with IgA Nephropathy. Kidney and Blood Pressure Research, 2017. 42(3): p. 541-552. |
| 79. Yang X, R Wei, Y Wang, et al. Decreased Serum C3 Levels in Immunoglobulin A (IgA) Nephropathy with Chronic Kidney Disease: A Propensity Score Matching Study. Med Sci Monit, 2017. 23: p. 673-81. |
| 80. Yang M, J Xie, Y Ouyang, et al. ABO blood type is associated with renal outcomes in patients with IgA nephropathy. Oncotarget, 2017. 8(43): p. 73603-73612. |
| 81. Wang W, G Li, D Hong, et al. Replication of genome-wide association study identified seven susceptibility genes, affirming the effect of rs2856717 on renal function and poor outcome of IgA nephropathy. Nephrology, 2017. 22(10): p. 811-817. |
| 82. Sevillano AM, E Gutiérrez, C Yuste, et al. Remission of hematuria improves renal survival in IgA nephropathy. Journal of the American Society of Nephrology, 2017. 28(10): p. 3089-3099. |
| 83. Matsukuma Y, K Masutani, S Tanaka, et al. A J-shaped association between serum uric acid levels and poor renal survival in female patients with IgA nephropathy. Hypertension Research, 2017. 40(3): p. 291-297. |
| 84. Lv J, H Zhang, MG Wong, et al. Effect of Oral Methylprednisolone on Clinical Outcomes in Patients With IgA Nephropathy: The TESTING Randomized Clinical Trial. Jama, 2017. 318(5): p. 432-42. |
| 85. Liu J, S Duan, P Chen, et al. Development and validation of a prognostic nomogram for IgA nephropathy. Oncotarget, 2017. 8(55): p. 94371-94381. |
| 86. Haas M, JC Verhave, ZH Liu, et al. A multicenter study of the predictive value of crescents in IgA nephropathy. Journal of the American Society of Nephrology, 2017. 28(2): p. 691-701. |
| 87. Guo W, L Zhu, S Meng, et al. Mannose-Binding Lectin Levels Could Predict Prognosis in IgA Nephropathy. J Am Soc Nephrol, 2017. 28(11): p. 3175-81. |
| 88. Woo KT, CC Lim, MWY Foo, et al. 30-year follow-up study of IgA nephritis in a Southeast Asian population: An evaluation of the Oxford histological classification. Clinical Nephrology, 2016. 86(5): p. 270-278. |
| 89. Tanaka S, T Ninomiya, R Katafuchi, et al. The effect of renin–angiotensin system blockade on the incidence of end-stage renal disease in IgA nephropathy. Clinical and Experimental Nephrology, 2016. 20(5): p. 689-698. |
| 90. Sarcina C, C Tinelli, F Ferrario, et al. Corticosteroid treatment influences TA-Proteinuria and renal survival in IgA nephropathy. PLoS ONE, 2016. 11(7). |
| 91. Ruggajo P, E Svarstad, S Leh, et al. Low birth weight and risk of progression to end stage renal disease in IgA nephropathy - A retrospective registry-based cohort study. PLoS ONE, 2016. 11(4). |
| 92. Ouyang Y, J Xie, M Yang, et al. Underweight Is an Independent Risk Factor for Renal Function Deterioration in Patients with IgA Nephropathy. PLoS One, 2016. 11(9). |
| 93. Kaneko Y, K Yoshita, E Kono, et al. Extracapillary proliferation and arteriolar hyalinosis are associated with long-term kidney survival in IgA nephropathy. Clinical and Experimental Nephrology, 2016. 20(4): p. 569-577. |
| 94. Kamei K, R Harada, R Hamada, et al. Proteinuria during Follow-Up Period and Long-Term Renal Survival of Childhood IgA Nephropathy. PLoS One, 2016. 11(3). |
| 95. Hoshino J, T Fujii, J Usui, et al. Renal outcome after tonsillectomy plus corticosteroid pulse therapy in patients with immunoglobulin A nephropathy: results of a multicenter cohort study. Clinical and Experimental Nephrology, 2016. 20(4): p. 618-627. |
| 96. Chakera A, C MacEwen, SS Bellur, et al. Prognostic value of endocapillary hypercellularity in IgA nephropathy patients with no immunosuppression. Journal of Nephrology, 2016. 29(3): p. 367-375. |
| 97. Yuan Y, Q Wang, Z Ni, et al. Long-term kidney survival analyses in IgA nephropathy patients under steroids therapy: a case control study. J Transl Med, 2015. 13. |
| 98. Yuan Y, X Che, Z Ni, et al. Association of Relapse with Renal Outcomes under the Current Therapy Regimen for IgA Nephropathy: A Multi-Center Study. PLoS One, 2015. 10(9). |
| 99. Tanaka S, T Ninomiya, K Masutani, et al. Prognostic impact of serum bilirubin level on long-term renal survival in IgA nephropathy. Clinical and Experimental Nephrology, 2015. 19(6): p. 1062-1070. |
| 100. Sato R, K Joh, A Komatsuda, et al. Validation of the Japanese histologic classification 2013 of immunoglobulin A nephropathy for prediction of long-term prognosis in a Japanese single-center cohort. Clinical and Experimental Nephrology, 2015. 19(3): p. 411-418. |
| 101. Ruan Y, W Chen, Z Li, et al. Role of immunosuppressive therapy and predictors of therapeutic effectiveness and renal outcome in IgA nephropathy with proteinuria. Archives of Medical Science, 2015. 11(2): p. 332-339. |
| 102. Rhee H, N Shin MJ Shin, et al. High serum and urine neutrophil gelatinaseassociated lipocalin levels are independent predictors of renal progression in patients with immunoglobulin A nephropathy. Korean J Intern Med, 2015. 30(3): p. 354-61. |
| 103. Moriyama T, M Itabashi, T Takei, et al. High uric acid level is a risk factor for progression of IgA nephropathy with chronic kidney disease stage G3a. Journal of Nephrology, 2015. 28(4): p. 451-456. |
| 104. Liu M, Y Chen, J Zhou, et al. Implication of Urinary Complement Factor H in the Progression of Immunoglobulin A Nephropathy. PLoS One, 2015. 10(6). |
| 105. Cheungpasitporn W, SH Nasr, C Thongprayoon, et al. Primary IgA nephropathy in elderly patients. Nephrology, 2015. 20(6): p. 419-425. |
| 106. Arroyo AH, AS Bomback, B Butler, et al. Predictors of outcome for severe IgA nephropathy in a multi-ethnic U.S. cohort. Clinical Nephrology, 2015. 84(3): p. 145-155. |
| 107. Park KS, SH Han, JH Kie, et al. Comparison of the Haas and the Oxford classifications for prediction of renal outcome in patients with IgA nephropathy. Human Pathology, 2014. 45(2): p. 236-243. |
| 108. Nam KH, JH Kie, MJ Lee, et al. Optimal Proteinuria Target for Renoprotection in Patients with IgA Nephropathy. PLoS One, 2014. 9(7). |
| 109. Moriyama T, K Tanaka, C Iwasaki, et al. Prognosis in IgA Nephropathy: 30-Year Analysis of 1,012 Patients at a Single Center in Japan. PLoS One, 2014. 9(3). |
| 110. Maixnerova D, M Neprasova, J Skibova, et al. IgA nephropathy in Czech patients - Are we able reliably predict the outcome? Kidney and Blood Pressure Research, 2014. 39(6): p. 555-562. |
| 111. Liu Y, X Ma, J Lv, et al. Risk factors for pregnancy outcomes in patients with IGA nephropathy: A matched cohort study. American Journal of Kidney Diseases, 2014. 64(5): p. 730-736. |
| 112. Li X, Y Liu, J Lv, et al. Progression of IgA Nephropathy under Current Therapy Regimen in a Chinese Population. Clin J Am Soc Nephrol, 2014. 9(3): p. 484-9. |
| 113. Lee H, JH Hwang, JH Paik, et al. Long-term prognosis of clinically early IgA nephropathy is not always favorable. BMC Nephrol, 2014. 15: p. 94. |
| 114. Le W, S Liang, H Chen, et al. Long-term outcome of IgA nephropathy patients with recurrent macroscopic hematuria. American journal of nephrology, 2014. 40(1): p. 43-50. |
| 115. Kovács T, T Vas, CP Kövesdy, et al. Effect of tonsillectomy and its timing on renal outcomes in Caucasian IgA nephropathy patients. International Urology and Nephrology, 2014. 46(11): p. 2175-2182. |
| 116. Espinosa M, R Ortega, M Sánchez, et al. Association of C4d Deposition with Clinical Outcomes in IgA Nephropathy. Clin J Am Soc Nephrol, 2014. 9(5): p. 897-904. |
| 117. Coppo R, S Troyanov, S Bellur, et al. Validation of the Oxford classification of IgA nephropathy in cohorts with different presentations and treatments. Kidney Int, 2014. 86(4): p. 828-36. |
| 118. Zhang J, C Wang, Y Tang, et al. Serum immunoglobulin A/C3 ratio predicts progression of immunoglobulin A nephropathy. Nephrology, 2013. 18(2): p. 125-131. |
| 119. Hu Y, Huang Z, Cao Q, et al. Clinical significance of massive proteinuria in primary IgA nephropathy with and without nephrotic syndrome: a single center cohort study [J]. Ren Fail, 2023, 45(2): 2267138. |
| 120. Knoop T, BE Vikse, E Svarstad, et al. Mortality in patients with IgA nephropathy. American Journal of Kidney Diseases, 2013. 62(5): p. 883-890. |
| 121. Barbour SJ, DC Cattran, SJ Kim, et al. Individuals of Pacific Asian origin with IgA nephropathy have an increased risk of progression to end-stage renal disease. Kidney International, 2013. 84(5): p. 1017-1024. |
| 122. Zhao N, P Hou, J Lv, et al. The level of galactose-deficient IgA1 in the sera of patients with IgA nephropathy is associated with disease progression. Kidney Int, 2012. 82(7): p. 790-6. |
| 123. Shi Y, W Chen, D Jalal, et al. Clinical Outcome of Hyperuricemia in IgA Nephropathy: A Retrospective Cohort Study and Randomized Controlled Trial. Kidney Blood Press Res, 2012. 35(3): p. 153-60. |
| 124. Lundberg S, AR Qureshi, S Olivecrona, et al, FGF23, Albuminuria, and Disease Progression in Patients with Chronic IgA Nephropathy. Clin J Am Soc Nephrol, 2012. 7(5): p. 727-34. |
| 125. Varughese S, S Alexander, S Roy, et al. Three-year clinical outcomes of the first South-Asian prospective longitudinal observational IgA nephropathy cohort (GRACE-IgANI). Kidney Diseases, 2021. 7(SUPPL 1): p. 81-82. |
| 126. Lee H, DK Kim, KH Oh, et al. Mortality of IgA Nephropathy Patients: A Single Center Experience over 30 Years. PLoS One, 2012. 7(12). |
| 127. Le WB, SS Liang, YL Hu, et al. Long-term renal survival and related risk factors in patients with IgA nephropathy: Results from a cohort of 1155 cases in a Chinese adult population. Nephrology Dialysis Transplantation, 2012. 27(4): p. 1479-1485. |
| 128. Gutiérrez E, I Zamora, JA Ballarín, et al. Long-Term Outcomes of IgA Nephropathy Presenting with Minimal or No Proteinuria. J Am Soc Nephrol, 2012. 23(10): p. 1753-60. |
| 129. Chou YH, YC Lien, FC Hu, et al, Clinical Outcomes and Predictors for ESRD and Mortality in Primary GN. Clin J Am Soc Nephrol, 2012. 7(9): p. 1401-8. |
| 130. Bjørneklett R, BE Vikse, L Bostad, et al, Long-term risk of ESRD in IgAN; Validation of Japanese prognostic model in a Norwegian cohort. Nephrology Dialysis Transplantation, 2012. 27(4): p. 1485-1491. |
| 131. Katafuchi R, T Ninomiya, M Nagata, et al. Validation Study of Oxford Classification of IgA Nephropathy: The Significance of Extracapillary Proliferation. Clin J Am Soc Nephrol, 2011. 6(12): p. 2806-13. |
| 132. Alamartine E, C Sauron, B Laurent, et al. The Use of the Oxford Classification of IgA Nephropathy to Predict Renal Survival. Clin J Am Soc Nephrol, 2011. 6(10): p. 2384-8. |
| 133. Walsh M, A Sar, D Lee, et al. Histopathologic Features Aid in Predicting Risk for Progression of IgA Nephropathy. Clin J Am Soc Nephrol, 2010. 5(3): p. 425-30. |
| 134. Chin HJ, HJ Cho, TW Lee, et al. The Mildly Elevated Serum Bilirubin Level is Negatively Associated with the Incidence of End Stage Renal Disease in Patients with IgA Nephropathy. J Korean Med Sci, 2009. 24(Suppl 1): p. S22-9. |
| 135. Yata N, K Nakanishi, Y Shima, et al. Improved renal survival in Japanese children with IgA nephropathy. Pediatr Nephrol, 2008. 23(6): p. 905-12. |
| 136. Katafuchi R, T Ninomiya, T Mizumasa, et al. The improvement of renal survival with steroid pulse therapy in IgA nephropathy. Nephrol Dial Transplant, 2008. 23(12): p. 3915-20. |
| 137. Berthoux FC, P Berthoux, C Mariat, et al. CC-chemokine receptor five gene polymorphism in primary IgA nephropathy: The 32 bp deletion allele is associated with late progression to end-stage renal failure with dialysis. Kidney International, 2006. 69(3): p. 565-572. |
| 138. Panzer U, A Schneider, OM Steinmetz, et al. The chemokine receptor 5 Δ32 mutation is associated with increased renal survival in patients with IgA nephropathy. Kidney International, 2005. 67(1): p. 75-81. |
| 139. Nozawa R, J Suzuki, A Takahashi, et al. Clinicopathological features and the prognosis of IgA nephropathy in Japanese children on long-term observation. Clinical Nephrology, 2005. 64(3): p. 171-179. |
| 140. Descamps-Latscha B, V Witko-Sarsat, T Nguyen-Khoa, et al. Early prediction of IgA nephropathy progression: Proteinuria and AOPP are strong prognostic markers. Kidney International, 2004. 66(4): p. 1606-1612. |
| 141. Li PKT, KKL Ho, CC Szeto, et al. Prognostic indicators of IgA nephropathy in the Chinese - Clinical and pathological perspectives. Nephrology Dialysis Transplantation, 2002. 17(1): p. 64-69. |
| 142. Donadio JV, JP Grande, EJ Bergstralh, et al. The long-term outcome of patients with IgA nephropathy treated with fish oil in a controlled trial. Mayo Nephrology Collaborative Group. Journal of the American Society of Nephrology : JASN, 1999. 10(8): p. 1772‐1777. |
| 143. Frimat L, S Briançon, D Hestin, et al. IgA nephropathy: Prognostic classification of end-stage renal failure. Nephrology Dialysis Transplantation, 1997. 12(12): p. 2569-2575. |
| 144. Frimat L, D Hestin, B Aymard, et al. IgA nephropathy in patients over 50 years of age: A multicentre, prospective study. Nephrology Dialysis Transplantation, 1996. 11(6): p. 1043-1047. |
| 145. Kang SW, KH Choi, JH Park, et al. Prognostic factors and renal survival rates in IgA nephropathy. Yonsei medical journal, 1995. 36(1): p. 45-52. |
| 146. Woo KT, KS Wong, YK Lau, et al. Hypertension in IgA nephropathy. Annals of the Academy of Medicine, Singapore, 1988. 17(4): p. 583-588. |
| 147. Xu X, X Huang, Y Chen, et al. The role of urine IgG in the progression of IgA nephropathy with a high proportion of global glomerulosclerosis. Int Urol Nephrol, 2022. 54(2): p. 323-330. |
| 148. Oh T R, H S Choi, S W Oh, et al. Association between the progression of immunoglobulin A nephropathy and a controlled status of hypertension in the first year after diagnosis. Korean J Intern Med, 2022. 37(1): p. 146-153. |
| 149. Lv J, M G Wong, M A Hladunewich, et al. Effect of Oral Methylprednisolone on Decline in Kidney Function or Kidney Failure in Patients With IgA Nephropathy: The TESTING Randomized Clinical Trial. Jama, 2022. 327(19): p. 1888-1898. |
| 150. Haaskjold Y L, R Bjørneklett, L Bostad, et al. Utilizing the MEST score for prognostic staging in IgA nephropathy. BMC Nephrol, 2022. 23(1): p. 26. |
| 151. Yang X, F Ma, M Bai, et al. The prognostic effect of immunosuppressive therapy in IgA nephropathy with stage 3 or 4 chronic kidney disease. Ren Fail, 2021. 43(1): p. 1180-1187. |
| 152. Tan L, Y Tang, G Q Pei, et al. Mesangial IgM deposition predicts renal outcome in patients with IgA nephropathy: a multicenter, observational study. Clin Exp Med, 2021. 21(4): p. 599-610. |
| 153. Pitcher D, F Braddon, B Hendry, et al. Long-Term Outcomes in IgA Nephropathy. Clinical journal of the American Society of Nephrology : CJASN, 2023. |
| 154. Gadola L, M J Cabrera, M Garau, et al. Long-term follow-up of an IgA nephropathy cohort: outcomes and risk factors. Renal Failure, 2023. 45(1). |
| 155. Zhu B, W H Liu, Y Lin, et al. Renal Interstitial Inflammation Predicts Nephropathy Progression in IgA Nephropathy: A Two-Center Cohort Study. American Journal of Nephrology, 2022. 53(6): p. 455-469. |
| 156. Weng M, J Lin, Y Chen, et al. Time-Averaged Hematuria as a Prognostic Indicator of Renal Outcome in Patients with IgA Nephropathy. Journal of Clinical Medicine, 2022. 11(22). |
| 157. Qin A, J Tan, S Wang, et al. Triglyceride–Glucose Index May Predict Renal Survival in Patients with IgA Nephropathy. Journal of Clinical Medicine, 2022. 11(17). |
| 158. Itami S, T Moriyama, Y Miyabe, et al. A Novel Scoring System Based on Oxford Classification Indicating Steroid Therapy Use for IgA Nephropathy. Kidney International Reports, 2022. 7(1): p. 99-107. |

Appendix E Sample size of the top20 countries included in the review

| Country | Sample size |
| --- | --- |
| China | 52202 |
| Japan | 14845 |
| Korea | 11582 |
| Sweden | 3802 |
| United Kingdom | 3716 |
| America | 3331 |
| Norway | 2493 |
| Italy | 1035 |
| France | 923 |
| Spain | 659 |
| Czech Republic | 520 |
| Greece | 457 |
| Germany | 352 |
| Hungary | 264 |
| Singapore | 253 |
| Romania | 248 |
| Uruguay | 241 |
| Brazil | 229 |
| India | 195 |
| Canada | 146 |

Appendix F. PRISMA checklist.

| **Section and Topic** | **Item #** | **Checklist item** | **Location where item is reported** |
| --- | --- | --- | --- |
| **TITLE** | | |  |
| Title | 1 | Identify the report as a systematic review. | 1 |
| **ABSTRACT** | | |  |
| Abstract | 2 | See the PRISMA 2020 for Abstracts checklist. | 2-3 |
| **INTRODUCTION** | | |  |
| Rationale | 3 | Describe the rationale for the review in the context of existing knowledge. | 4-5 |
| Objectives | 4 | Provide an explicit statement of the objective(s) or question(s) the review addresses. | 6 |
| **METHODS** | | |  |
| Eligibility criteria | 5 | Specify the inclusion and exclusion criteria for the review and how studies were grouped for the syntheses. | 7-9 |
| Information sources | 6 | Specify all databases, registers, websites, organisations, reference lists and other sources searched or consulted to identify studies. Specify the date when each source was last searched or consulted. | 7 |
| Search strategy | 7 | Present the full search strategies for all databases, registers and websites, including any filters and limits used. | 7 |
| Selection process | 8 | Specify the methods used to decide whether a study met the inclusion criteria of the review, including how many reviewers screened each record and each report retrieved, whether they worked independently, and if applicable, details of automation tools used in the process. | 7-8 |
| Data collection process | 9 | Specify the methods used to collect data from reports, including how many reviewers collected data from each report, whether they worked independently, any processes for obtaining or confirming data from study investigators, and if applicable, details of automation tools used in the process. | 7-9 |
| Data items | 10a | List and define all outcomes for which data were sought. Specify whether all results that were compatible with each outcome domain in each study were sought (e.g. for all measures, time points, analyses), and if not, the methods used to decide which results to collect. | 7-9 |
|  | 10b | List and define all other variables for which data were sought (e.g. participant and intervention characteristics, funding sources). Describe any assumptions made about any missing or unclear information. | 7-9 |
| Study risk of bias assessment | 11 | Specify the methods used to assess risk of bias in the included studies, including details of the tool(s) used, how many reviewers assessed each study and whether they worked independently, and if applicable, details of automation tools used in the process. | 7-9 |
| Effect measures | 12 | Specify for each outcome the effect measure(s) (e.g. risk ratio, mean difference) used in the synthesis or presentation of results. | 8-9 |
| Synthesis methods | 13a | Describe the processes used to decide which studies were eligible for each synthesis (e.g. tabulating the study intervention characteristics and comparing against the planned groups for each synthesis (item #5)). | 8-9 |
|  | 13b | Describe any methods required to prepare the data for presentation or synthesis, such as handling of missing summary statistics, or data conversions. | 8-9 |
|  | 13c | Describe any methods used to tabulate or visually display results of individual studies and syntheses. | 8-9 |
|  | 13d | Describe any methods used to synthesize results and provide a rationale for the choice(s). If meta-analysis was performed, describe the model(s), method(s) to identify the presence and extent of statistical heterogeneity, and software package(s) used. | 8-9 |
|  | 13e | Describe any methods used to explore possible causes of heterogeneity among study results (e.g. subgroup analysis, meta-regression). | 9 |
|  | 13f | Describe any sensitivity analyses conducted to assess robustness of the synthesized results. | - |
| Reporting bias assessment | 14 | Describe any methods used to assess risk of bias due to missing results in a synthesis (arising from reporting biases). | - |
| Certainty assessment | 15 | Describe any methods used to assess certainty (or confidence) in the body of evidence for an outcome. | - |
| **RESULTS** | | |  |
| Study selection | 16a | Describe the results of the search and selection process, from the number of records identified in the search to the number of studies included in the review, ideally using a flow diagram. | 9 |
|  | 16b | Cite studies that might appear to meet the inclusion criteria, but which were excluded, and explain why they were excluded. | 9 |
| Study characteristics | 17 | Cite each included study and present its characteristics. | 9 |
| Risk of bias in studies | 18 | Present assessments of risk of bias for each included study. | 9 |
| Results of individual studies | 19 | For all outcomes, present, for each study: (a) summary statistics for each group (where appropriate) and (b) an effect estimate and its precision (e.g. confidence/credible interval), ideally using structured tables or plots. | 10-12 |
| Results of syntheses | 20a | For each synthesis, briefly summarise the characteristics and risk of bias among contributing studies. | 17 |
|  | 20b | Present results of all statistical syntheses conducted. If meta-analysis was done, present for each the summary estimate and its precision (e.g. confidence/credible interval) and measures of statistical heterogeneity. If comparing groups, describe the direction of the effect. | - |
|  | 20c | Present results of all investigations of possible causes of heterogeneity among study results. | - |
|  | 20d | Present results of all sensitivity analyses conducted to assess the robustness of the synthesized results. | - |
| Reporting biases | 21 | Present assessments of risk of bias due to missing results (arising from reporting biases) for each synthesis assessed. | - |
| Certainty of evidence | 22 | Present assessments of certainty (or confidence) in the body of evidence for each outcome assessed. | - |
| **DISCUSSION** | | |  |
| Discussion | 23a | Provide a general interpretation of the results in the context of other evidence. | 12-17 |
|  | 23b | Discuss any limitations of the evidence included in the review. | 17 |
|  | 23c | Discuss any limitations of the review processes used. | 17 |
|  | 23d | Discuss implications of the results for practice, policy, and future research. | 12-17 |
| **OTHER INFORMATION** | | |  |
| Registration and protocol | 24a | Provide registration information for the review, including register name and registration number, or state that the review was not registered. | 7 |
|  | 24b | Indicate where the review protocol can be accessed, or state that a protocol was not prepared. | - |
|  | 24c | Describe and explain any amendments to information provided at registration or in the protocol. | - |
| Support | 25 | Describe sources of financial or non-financial support for the review, and the role of the funders or sponsors in the review. | 18-19 |
| Competing interests | 26 | Declare any competing interests of review authors. | 19 |
| Availability of data, code and other materials | 27 | Report which of the following are publicly available and where they can be found: template data collection forms; data extracted from included studies; data used for all analyses; analytic code; any other materials used in the review. | 18 |

*From:*  Page MJ, McKenzie JE, Bossuyt PM, Boutron I, Hoffmann TC, Mulrow CD, et al. The PRISMA 2020 statement: an updated guideline for reporting systematic reviews. BMJ 2021;372:n71. doi: 10.1136/bmj.n71

For more information, visit: <http://www.prisma-statement.org/>

Appendix G Estimated renal survival at 3 years, 5 years of IgAN after excluding cohorts with enrollment periods longer than 10 years.


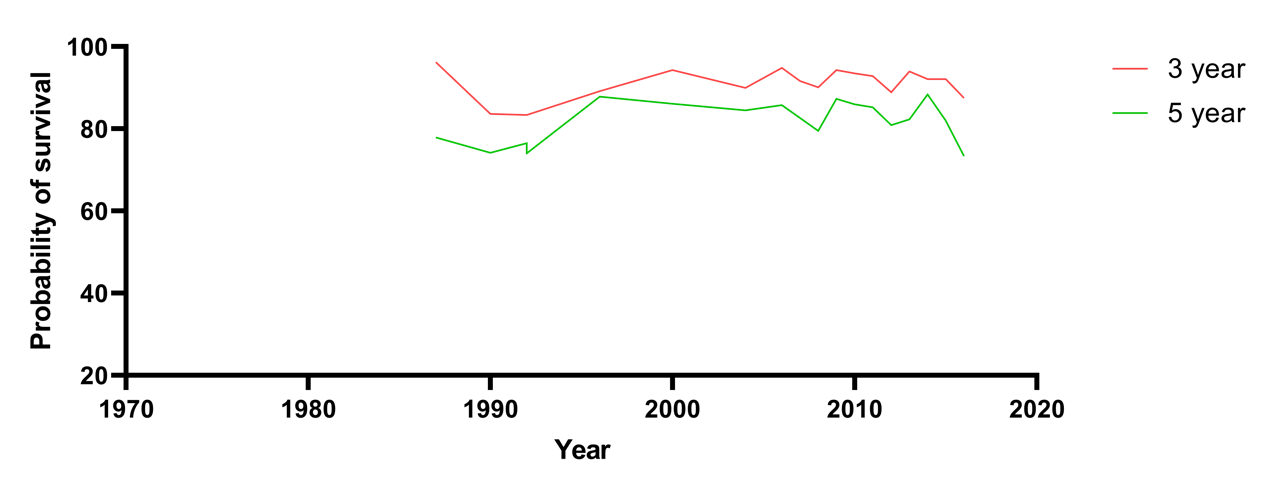


Appendix H The 3-year, 5-year and 10-year median survival in developed countries and developing countries

| country | 3 year % | 5 year % | 10 year % |
| --- | --- | --- | --- |
| developed countries | 96.25(94.72,96.25) | 93.08(89.16,93.40) | 84.39(78.06,85.62) |
| developing countries | 93.76(90.95,94.41) | 88.91(82.12,90.11) | 73.04(62.76,78.51) |
| *P* | <0.001 | <0.001 | <0.001 |
